# Valgus bracing can relocate cartilage contact pressure away from bone marrow lesions and full-thickness cartilage defects in varus-malaligned medial knee osteoarthritis

**DOI:** 10.1016/j.ocarto.2026.100871

**Published:** 2026-08-28

**Authors:** Scott C. Starkey, Amir Esrafilian, David J. Saxby, Win Min Oo, Laura E. Diamond, Rami K. Korhonen, David J. Hunter, Michelle Hall

**Affiliations:** a Centre for Health, Exercise and Sports Medicine, Department of Physiotherapy, The University of Melbourne, Melbourne, Australia; b Department of Technical Physics, University of Eastern Finland, Kuopio, Finland; c Australian Centre for Precision Health and Technology (PRECISE), Griffith University, Gold Coast, Australia; d School of Human Sciences, The University of Western Australia, Crawley, Australia; e School of Allied Health, Sport and Social Work, Griffith University, Gold Coast, Australia; f Department of Physical Medicine and Rehabilitation, Mandalay General Hospital, University of Medicine, Mandalay, Mandalay, Myanmar; g Sydney Musculoskeletal Centre, Kolling Institute, Faculty of Medicine and Health, The University of Sydney, Sydney, Australia; h Clinical Research Center, Kuopio University Hospital, Wellbeing Services County of North Savo, Kuopio, Finland; i Sydney School of Health Sciences, The University of Sydney, Sydney, Australia; j Musculoskeletal Research Hub, Charles Perkins Centre, The University of Sydney, Sydney, Australia

**Keywords:** Biomechanics, Mechanisms, Cartilage, Osteochondral, bone marrow, Musculoskeletal modelling, Conservative, Therapeutic, Pain

## Abstract

**Objective:**

To examine whether valgus bracing reduces cartilage contact pressure atop structural sources of weight-bearing pain in varus-malaligned medial knee osteoarthritis.

**Design:**

An exploratory secondary analysis of a within-participant longitudinal study involving an 8-week valgus brace intervention.

**Methods:**

Baseline biomechanical data and 8-week change scores in the KOOS pain subscale from participants with ≥1 bone marrow lesion (BML) on the medial tibia were analysed (n = 21). Magnetic resonance imaging (MRI) assessed medial tibial BMLs and full-thickness cartilage defects. A 12 degree-of-freedom MRI-informed knee neuromusculoskeletal model quantified cartilage pressure exerted atop BMLs and cartilage defects. Peak pressure magnitude (MPa) atop BMLs and defects was compared between braced and unbraced walking using paired t-tests. Responder rate (did/did not reduce pressure atop BMLs/defects, and ≥MCID KOOS pain) was examined using Fisher’s exact test.

**Results:**

Cartilage contact pressure was exerted atop BML(s) during unbraced walking for 15 of 21 (71%) participants; and in 9 of 11 (82%) participants who had cartilage defects. Braced walking reduced peak magnitude pressure atop BMLs by 13% (−1.2 MPa [95%CI −2.0, −0.4], p = 0.008) and defects by 18% (−1.9 MPa [95%CI −3.3, −0.5], p = 0.013). Participants who reduced peak pressure atop a BML and/or cartilage defect were more likely to exceed the KOOS MCID than those who did not (risk difference: 60 percentage points [95%CI 17, 84], p = 0.011).

**Conclusions:**

Valgus bracing reduced cartilage contact pressure atop BMLs and defects among those who loaded these lesions. This biomechanical response was associated with a pain improvement, a hypothesis-generating finding that warrants further examination.

## 1 Introduction

Pain is the omnipresent feature of symptomatic knee osteoarthritis (OA), with pain relief being the primary reason patients seek medical care [1]. Valgus bracing is an appealing biomechanical intervention for patients with medial knee OA and varus malalignment. However, clinical practice guidelines provide conflicting recommendations regarding valgus brace use for medial knee OA [2] due to heterogeneous patient responses in clinical trials [3,4]. The heterogeneity in patient responses to valgus bracing could be reduced by identifying subgroups of patients who may benefit most from valgus bracing.

Knee OA pain typically occurs or is exacerbated during weight-bearing activities [5]. This suggests that mechanical loading of nociceptive (and other) structures within the knee may be a contributing mechanism to weight-bearing knee pain [6]. Of particular interest to weight-bearing knee OA pain is the subchondral bone, a richly innervated region of bone located immediately underneath the articulating tibiofemoral cartilages [7]. Bone marrow lesions (BMLs) are circumscribed lesions within subchondral bone that have been observed on magnetic resonance imaging (MRI) in up to 80% of patients with symptomatic OA [8]. Critically, greater semi-quantitative BML scores of the tibiofemoral joint have been associated with greater weight-bearing pain; suggesting that BMLs at the knee may be a key contributor to biomechanically-induced knee OA pain [9]. Although cartilage does not contain pain receptors/fibres, full-thickness articular cartilage lesions denuded to subchondral bone have also been associated with prevalent and incident knee OA pain [10]. Therefore, biomechanical interventions such as valgus bracing that aim to reduce pressure atop BMLs and full-thickness cartilage defects may impart clinical benefit to those with knee OA by reducing pain during weight-bearing activities.

Neuro-musculoskeletal models are valuable tools with which to study the effect of valgus bracing on knee mechanics, as they consider both the applied loads (i.e., brace torque) and the body’s neuromuscular patterns in response to these brace-generated torques [11]. Of particular interest are neuro-musculoskeletal models capable of simulating tissue-level knee loading [12,13]. These models use medical imaging, such as MRI, to create subject-specific 3D models of bone, ligament, and cartilage, including spatially varying cartilage thickness and focal lesions [12,13]. Laboratory-acquired 3D motion capture and electromyography data are then used to drive model simulations of 3D tibiofemoral cartilage contact mechanics, including the magnitude and location of cartilage pressure [12,13]. Traditionally, these models were time- and resource-intensive to create, but recent developments in convolutional neural networks and analysis pipelines have made it possible to implement these models rapidly in large cohorts [[14], [15], [16]].

In our previous study, valgus braced walking reduced peak medial tibial cartilage contact pressure by 11%, and shifted the mean pressure location upon the medial tibial cartilage laterally (by 5% of cartilage width) and posteriorly (by 2% of cartilage length) [17]. These novel findings suggest valgus bracing can alter both the magnitude and location of pressure upon the medial tibial cartilage. To extend the potential clinical relevance of these findings, it is important to determine whether load redistribution reduces pressure atop structures reported to be sources of weight-bearing pain (i.e., BMLs, full-thickness cartilage lesions [9,10]).

Therefore, in people who have varus-malaligned medial tibiofemoral OA and BMLs, the present exploratory study aimed to (1) determine the immediate effect of valgus bracing on medial tibial cartilage contact pressure atop BMLs and full-thickness cartilage defects (if present) during walking; and (2) examine the relationship between individual participant change scores in self-reported knee pain and cartilage pressure atop BMLs/full-thickness cartilage defects. It was hypothesised that valgus bracing would reduce pressure atop BMLs and full-thickness cartilage defects in participants who mechanically load them during walking; and that this biomechanical response would be associated with a clinical response (i.e., pain improvement). Establishing therapeutic mechanisms of action of valgus bracing may help clinicians identify which of their knee OA patients would benefit most from this intervention.

## 2 Method

An exploratory within-participant longitudinal study design was used to test the immediate effect of valgus braced walking on medial tibial contact pressure atop BMLs and full-thickness cartilage defects. This study was conducted and reported in accordance with STROBE guidelines [18] (Supplementary Table 1). Baseline (0 week) biomechanical data and MRI were obtained from a previous study that investigated the 8-week effects of a valgus brace on medial tibiofemoral joint contact force [19]. Longitudinal changes (week 8 minus week 0) in the pain subscale of the Knee Injury and Osteoarthritis Outcome Score (KOOS) [20] were obtained [19]. The study had ethical approval obtained from the Institutional Human Research Ethics Committee (#1853473) and was prospectively registered in the Australian and New Zealand Clinical Trials Registry (#12619000622101).

### 2.1 Participants

As this was an exploratory (hypothesis-generating) analysis, a formal priori sample size/power calculation was not performed. Instead, effect sizes (with 95% confidence intervals) were reported to support power calculations of future prospective studies.

Complete details of study procedures, including participant recruitment, have been published previously [19]. Twenty-eight volunteers were recruited from the community in Melbourne, Australia, between April–November 2019. Knee OA was classified using the American College of Rheumatology clinical and radiographic criteria [21]. Main inclusion criteria were (i) aged ≥50 years; (ii) radiographic tibiofemoral joint OA (Kellgren & Lawrence grade ≥2) [22]; (iii) radiographical varus malalignment (defined as an anatomic axis angle of <183° for males and <181° for females) [23,24]; (iv) knee pain on most days of the past month for >3 months; and (v) knee pain while walking ≥4 on a 10 point numerical rating scale over the week prior to, and at, baseline biomechanical testing. Main exclusion criteria were: (i) knee surgery over the past 6 months; (ii) planning to see an orthopaedic surgeon about a knee problem over the next 8 weeks; (iii) awaiting or planning any back or lower-limb surgery over the next 3 months; (iv) current or past (3 months) use of intra-articular or oral corticosteroids; (v) current or past (6 months) muscular or joint condition other than knee OA; (vi) systemic arthritis; (vii) current or past (6 months) use of, or intention to use in the next 8 weeks, a knee brace, walking stick or gait aid; (viii) body mass index >36 kg m^−2^; (ix) lateral joint space narrowing ≥ medial joint space narrowing; and (x) lateral osteophyte grade ≥ medial compartment osteophyte grade. Additional eligibility criteria were imposed for this secondary data analysis. Participants must have ≥1 BML located in the medial tibia, defined as a score of ≥1 on the ‘BML Size including cyst subscale’ on the MRI Osteoarthritis Knee Score (MOAKS) [25]. Furthermore, participants were excluded from analysis if their MRI data had excessive artifacts (i.e., limiting accurate visualization of knee cartilage or BMLs).

### 2.2 Procedures

**Fig. 1:**
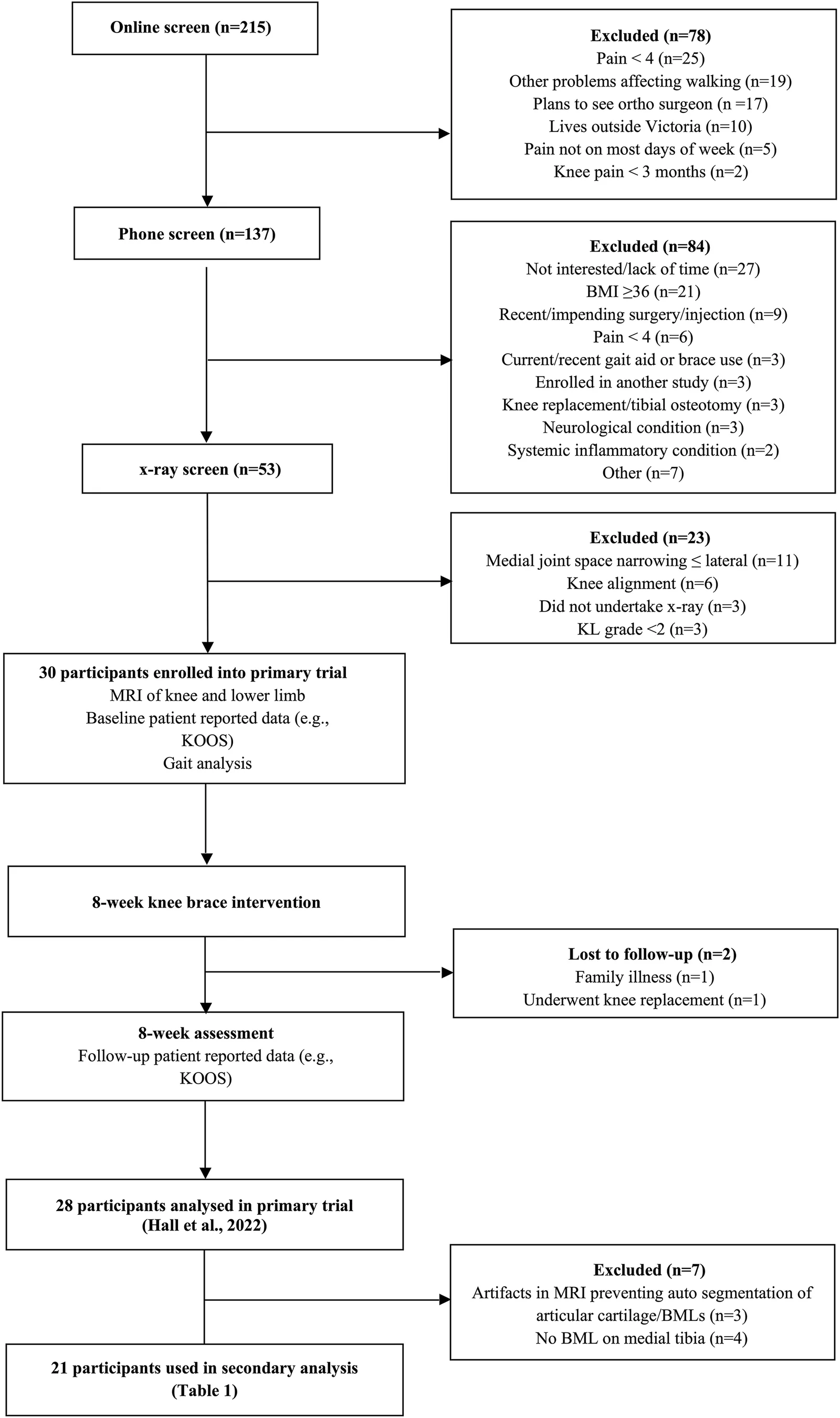
Flow-diagram outlining parent study participant recruitment, screening, exclusions/dropouts and endpoints, alongside secondary analysis exclusions (for current study).

A flow-diagram of study procedures, including participant recruitment, screening, and endpoints for secondary analysis is provided in Fig. 1. Volunteers were screened via an online survey followed by telephone screening to confirm their eligibility. Potentially eligible participants underwent a knee x-ray if they did not have their own knee x-ray within the past 12 months. If a participant had bilateral symptoms, the most symptomatic eligible knee was considered the study knee. Participant reported data were collected using REDCap at the beginning of the baseline (0 week) and follow-up (8 week) biomechanical testing sessions. Biomechanical data were collected by the same researcher (SCS) at the Department of Physiotherapy Human Movement Laboratory, University of Melbourne.

### 2.3 Brace intervention

An off-the-shelf valgus knee brace (Unloader One©, Össur, Reykjavik, Iceland) was evaluated. At the beginning of the 0-week biomechanical testing session, participants were fitted with the knee brace by a physiotherapist (SCS) who had been trained by the manufacturer and an orthotist. The tension on the superior and inferior dynamic force straps was set to alleviate or minimize the participant’s knee pain while walking when the SmartDosing™ dial was set to the middle (i.e., number 5). Once fitted, participants were then asked to walk with the brace for 10 min prior to biomechanical testing to ensure familiarization with the device.

At the conclusion of the week 0 biomechanical testing, participants were provided with a demonstration, written material, and instruction video of how to wear the brace and self-adjust the strap tension. Participants were instructed to gradually increase the use of the brace by 1–2 h per day until they were wearing the brace “whenever you are on your feet performing daily activities” for the next 8 weeks. If an adverse event or fitting issues occur with the brace, participants were encouraged to contact the study physiotherapist (SCS) for troubleshooting. If the problem could not be resolved, participants were seen face-to-face by the study physiotherapist and orthotist at the movement laboratory to refit the brace.

### 2.4 Data acquisition

#### 2.4.1 Biomechanical data

Participants completed six trials walking with and without the valgus brace in a block randomised order. Walking speed was self-selected and matched to ±5% across trials and conditions measured using photo-electric timing gates. A 12-camera 3D motion analysis system (Vicon MX, Oxford, UK) and two ground-embedded force plates (AMTI OR6, Massachusetts, USA) were used to collect kinematic (120 Hz) and ground reaction force (1200 Hz) data, respectively. Sixty-seven retro-reflective markers were placed on the participants’ skin according to the University of Western Australia marker set [26]. A 16-channel wireless electromyography (EMG) system (Noraxon, Arizona, USA) was used to record activation of twelve lower-limb muscles of the study leg at 1200 Hz in accordance with SENIAM guidelines [27]. Participants performed a series of maximum isometric voluntary contractions to elicit maximal EMG amplitudes for each of the twelve instrumented muscles [19].

#### 2.4.2 MRI

Within the same day of the biomechanical testing, participants attended the Royal Children’s Hospital Medical Imaging Department for an MRI using a 3 T MRI machine (Siemens Medical Systems, Erlangen, German). The MRI sequence involved a 3D T_1_-weighted sagittal volumetric interpolated breath-hold examination (VIBE) of the study knee and a T_1_-weighted coronal sequence of the pelvis and bilateral lower-limbs. The MRI protocol is detailed in Supplementary Table 2.

#### 2.4.3 Self-reported knee pain

Self-reported knee pain was examined at baseline and after the 8-week brace intervention using the pain subscale of the Knee Injury and Osteoarthritis Outcome Score (KOOS) [20].

### 2.5 Data analysis

#### 2.5.1 Tibial cartilage contact pressure analysis

Detailed methodology behind the simulation of tibiofemoral cartilage contact pressure for this dataset is reported elsewhere [17] and in Supplementary Table 3. Briefly, a previously trained 3D nnU-Net artificial neural network produced 3D segmentations of knee cartilages, bones, ligaments, menisci, and patella/quadriceps tendons for each participant from their T_1_ VIBE MRI [14]. These 3D segmentations were then integrated into a generic full-body musculoskeletal model validated for walking [13,28,29] by morphing a template musculoskeletal model to the segmented geometries, including articulating surfaces and ligament insertion points [14]. The resulting model had 3 degrees-of-freedom at the hip, 12 degrees-of-freedom at the knee (three rotational and three translational at both the tibiofemoral and patellofemoral joints), and 1 degrees-of-freedom at the ankle. The motion of the knee model was constrained by 12 bundles of non-linear elastic ligaments and capsular structures [30]. Brace action was modelled as a pure abduction moment about the tibia body, which varied in magnitude as a function of the participants’ knee flexion/extension angle using data provided by the manufacturer.

Model musculotendon-unit parameters were calibrated using two arbitrarily selected unbraced and braced walking trials via the Calibrated EMG-Informed Neuromusculoskeletal modelling toolbox [31]. Knee kinematics and muscle forces were simultaneously estimated using the remaining four unbraced and braced walking trials not used during calibration via an EMG-informed version of the Concurrent Optimization of Muscle Activation and Kinematics toolbox in OpenSim (Version 4.5) [29,32,33]. Tibial cartilage contact pressure (MPa) was then estimated using a previously validated nonlinear elastic foundation contact algorithm via the Joint Articular Mechanics toolbox in OpenSim [[33], [34], [35]]. For this study, mean tibial cartilage contact pressure was examined, which relates to the central tendency of pressure on the cartilage [17]. The 2D spatial coordinate time series (anterior-posterior, medial-lateral) of the medial tibial cartilage centre of pressure, relative to the origin of the knee joint coordinate system, was also extracted.

#### 2.5.2 Bone marrow lesion and cartilage defect analysis

**Fig. 2:**
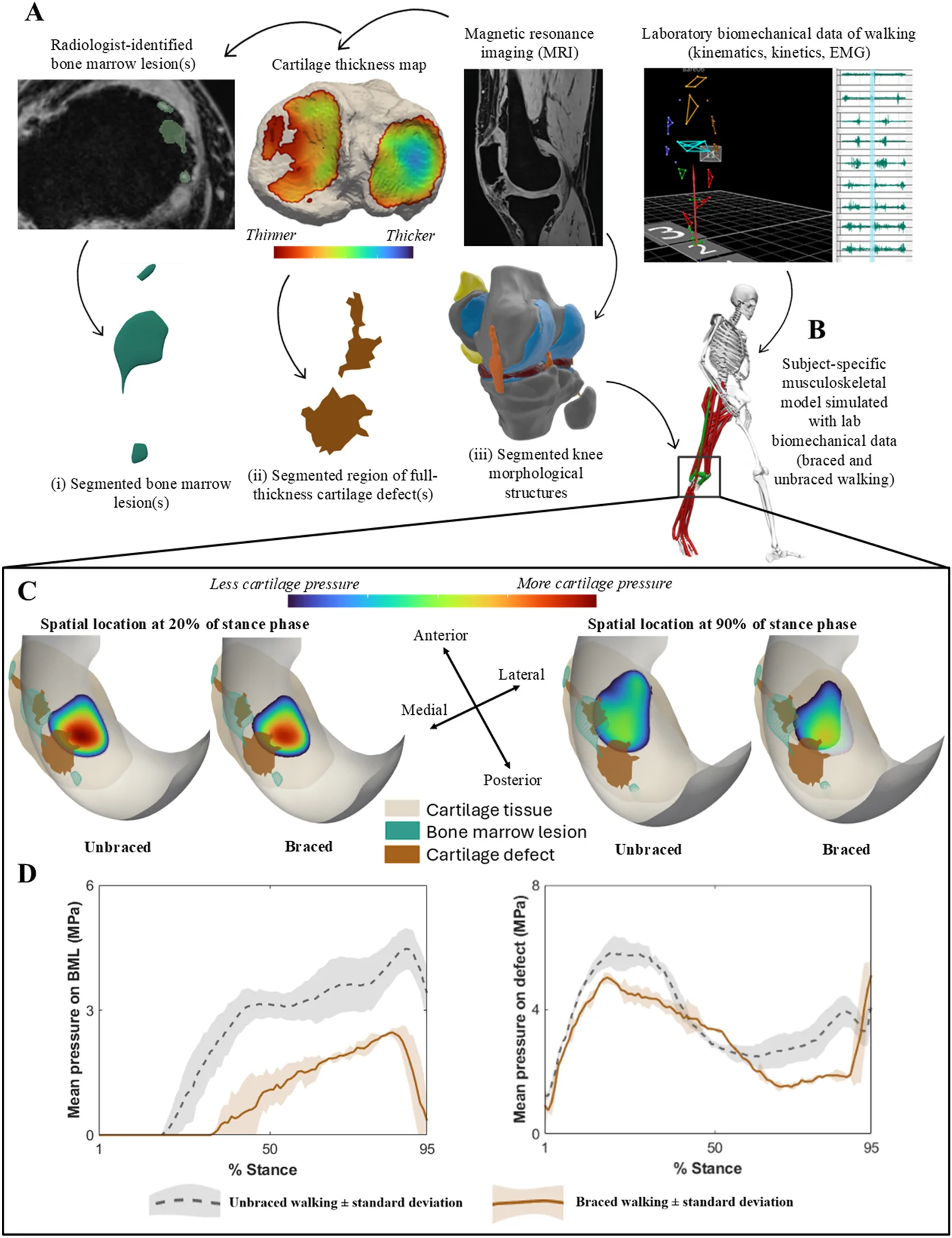
Flow diagram of BML and cartilage defect analysis process. (A) Knee MRIs are used to create (i) segmentations of bone marrow lesions (BMLs) identified by the trained researcher in MOAKS (WMO); (ii) segmentations of the region of full-thickness cartilage defects derived from cartilage thickness maps; and (iii) segmentations of knee morphological structures (bone, cartilage, ligaments, tendons), the latter of which are integrated into an existing full-body musculoskeletal model. (B) The subject-specific neuromusculoskeletal model is calibrated and then simulated using biomechanical data from unbraced and braced walking to estimate the time series of mean cartilage contact pressure atop the participants’ BML(s) or cartilage defect(s). (C) A spatial representation of cartilage contact pressure and BML/cartilage defect region for a participant’s medial tibia at 20% and 90% of stance for unbraced and braced walking. (D) Associated (0–95%) waveforms from the stance phase of walking showing the mean ± SD pressure atop the BML and cartilage defect. Abbreviations: MOAKS = MRI Osteoarthritis Knee Score; EMG = electromyography.

A flow-diagram outlining the creation and simulation of cartilage contact pressure atop BMLs and cartilage defects is provided (Fig. 2). For each participant, their tibial BMLs and full-thickness cartilage defects were assessed semi-quantitatively by a trained researcher (WMO) with seven years of experience in using the MOAKS instrument [25]. The same researcher selected the MRI axial slice from the T_1_ VIBE sequence where the BML was largest and manually segmented the perimeter of the BML using 3D Slicer software [36]. The largest slice was selected, as this as this best describes the 2D spatial coverage of the BML within the medial tibia. Where applicable, multiple BMLs were segmented within the same slice. Participant’s BML perimeters were then exported in stereolithography (STL) file format and transformed to the knee coordinate system using transformation matrices obtained during musculoskeletal model creation [37].

To analyse full-thickness defects of the medial tibial cartilage, thickness maps were created using Visualization Toolkit Python libraries [38] by casting rays from the subchondral bone normal to the surface and emanating from each vertex on the bone mesh surface [37]. If the ray had two intersections with the corresponding cartilage geometry (within an arbitrary range of ±1 cm), the distance between the two intersections was considered cartilage thickness, and its magnitude was assigned to the vertex on the bone from which the ray emanated. Thus, the cartilage thickness map had a spatial resolution equal to the number of bone vertices. All cartilage thickness values of 0 mm (i.e., full-thickness defect) were identified using custom Matlab scripts (version 2024b, MathWorks, Massachusetts, USA) and their spatial locations were exported as an STL file and transformed to the knee coordinate system in an identical manner to the BMLs (explained above).

#### 2.5.3 Analysis of tibial cartilage contact pressure atop BMLs and cartilage defects

Analysis of cartilage contact pressure atop medial tibial BMLs and full-thickness cartilage defects was performed in Matlab using a collision detection algorithm that found the union (mm^2^) between areas of medial tibial cartilage contact pressure and the areas of the BML or cartilage defect. The peak magnitude cartilage contact pressure (MPa) and cartilage contact pressure time-integral across stance (MPa·s) atop the BML/cartilage defect were then calculated.

### 2.6 Statistical analysis

Statistical analysis was conducted using Statistical Package for Social Science version 30 (IBM, New York, USA). Participant characteristics were described using means and standard deviations for continuous variables, or number and percentage for categorical variables.

#### 2.6.1 Statistical analysis for study aim 1

Dependent variables used to address study aim 1 were (1) peak magnitude cartilage contact pressure atop BML; (2) stance cartilage contact pressure time-integral atop BML; (3) peak magnitude cartilage contact pressure atop full-thickness cartilage defect; (4) stance cartilage contact pressure time-integral atop full-thickness cartilage defect; and (5) walking speed. Dependent variables were compared between independent variables (braced and unbraced walking) using paired t-tests with an alpha level set to 0.05. Normality of paired differences was confirmed using the Shapiro-Wilk test.

#### 2.6.2 Statistical analysis for study aim 2

Participant KOOS pain subscale scores were transformed to a 0–100 scale and then used to calculate change scores (week 8 minus week 0). Next, participant change scores (braced mins unbraced) were calculated for medial tibial cartilage contact pressure peak magnitude and pressure-time integral across stance atop (i) BMLs and (ii) cartilage defects. Graphical software (QtiPlot, IONDEV, Romania) generated sex-stratified scatter plots using the KOOS pain change scores as the X coordinate and the change scores for the tibial cartilage contact pressure variables (peak magnitude and pressure-time integral across stance) as the Y coordinates.

Participants were dichotomised by whether contact pressure atop the BML and/or defect was reduced during braced relative to unbraced walking. This biomechanical classification was applied separately for BMLs, cartilage defects, and a combined measure considering either structure, and repeated using both peak magnitude and stance time-integral pressure. Between-group differences (participants where bracing reduced pressure atop lesions vs those who did not) in KOOS pain change scores were compared using Welch’s t-tests with mean differences (95% CI) and Cohen’s d (95% CI) as the effect size. Furthermore, responder status (≥MCID KOOS pain) with risk differences (95% CI) was compared between groups using Fisher’s exact test.

A supplementary responder analysis was conducted to assess whether MRI presence of a weight-bearing BML and/or full-thickness cartilage defect alone could identify pain responders. Participants were dichotomised by presence of a BML and/or cartilage defect within the middle third of the medial tibial plateau on MOAKS (the primary tibiofemoral contact zone during walking [17]). Between-group differences (lesions present in middle third vs lesions present) in KOOS pain change scores were examined using Welch’s t-tests and responder status (≥MCID KOOS pain) via Fisher’s exact test, as described above. Furthermore, linear regression modelled the association between the KOOS pain change score (independent variable) and change in cartilage contact pressure atop BMLs, full-thickness cartilage defects, and the combined measure (BMLs and/or defects). Associations were reported as unstandardised regression coefficients (β) and 95% CI, alongside the coefficient of determination (R^2^).

To assess whether ceiling or floor effects influenced responder classification, we checked baseline and follow-up KOOS pain scores for values at the scale extremes (0 or 100), compared baseline scores between responders and non-responders using Welch’s *t*-test, and examined the correlation between baseline score and subsequent change using Pearson’s correlation coefficient.

## 3 Results

### 3.1 Cohort description

Data from 21 of the original 28 study participants were included in this secondary analysis (Table 1). Of the seven participants excluded, three (43%) had artifacts (noise) in their MRI preventing segmentation of articular cartilage, while four (57%) did not have any BML located within the medial tibia. The cohort had more males than females, were on average overweight to obese, had an equal distribution of mild, moderate, and severe OA, and reported a median pain during walking of 6 on the 10-points numerical rating scale for the week prior to baseline biomechanical testing.

**Table 1:** Participant characteristics.

Cohort, n	21
Age, yr	63.9 (4.7)
Male, n (%)	13 (62%)
Height, m	1.68 (0.11)
Weight, kg	84.6 (14.8)
Body mass index, kg⋅m^−2^	29.8 (3.7)
Unilateral symptoms, n (%)	10 (48%)
Duration of symptoms, yr	5.4 (4.7)
Most affected leg, right (%)	16 (76%)
Test leg dominant, yes (%)	19 (90%)
Knee alignment, degrees^a^	
Females	178.4 (2.7)
Males	178.0 (2.9)
Radiographic disease severity grade, n (%)^b^	
Grade 2	7 (33%)
Grade 3	7 (33%)
Grade 4	7 (33%)
Pain while walking over past week^c^	6 (1.7)
KOOS pain subscale	
Baseline	49 (13)
Follow-up (8 weeks)	69 (16)

∗Except where indicated, values are mean (standard deviation).

†KOOS: Knee injury and Osteoarthritis Outcome Score (scored 0–100 points, higher scores indicate less pain).

a Anatomic alignment, where varus alignment is < 181° for females and <183° for males.

b Kellgren-Lawrence grading system.

c Numerical rating scale (0 = no pain and 10 = worst pain possible).

Examination of cohort MOAKS scores showed the middle 1/3 of the medial tibia to have the largest number of BMLs and full-thickness cartilage defects, followed by the anterior 1/3, then posterior 1/3 (Table 2). The anterior and middle 1/3 of the tibia had similar prevalence of large (grade 2/3) BMLs and full-thickness defects, with the posterior 1/3 having minimal (Table 2). Quantitative analysis of medial tibial cartilage showed the anterior-lateral region to have the thinnest cartilage (1.0 ± 0.5 mm), followed by the antero-medial region (1.2 ± 0.5 mm) (Table 2). Mean BML cross-sectional area (as a percentage of total medial tibial cartilage area) was 11.1% greater than full-thickness defect cross-sectional area (BML: 16.2 ± 15.2 %, defect: 5.1 ± 6.6 %) (Table 2).

**Table 2:** Characteristics of bone marrow lesions (BML) and full-thickness cartilage defects for regions of the medial tibia.

Cohort, n	21
BML size (% of medial tibial cartilage area)	16.2 (15.2)
Full-thickness cartilage defect size (% of medial tibial cartilage area)^a^	5.1 (6.6)
MOAKS sub-scores	
BML size including cyst^b^	
Anterior 1/3	Grade 0: 7 (33.3%) Grade 1: 9 (42.9%) Grade 2: 5 (23.8%) Grade 3: 0 (0.0%)
Middle 1/3	Grade 0: 3 (14.3%) Grade 1: 13 (61.9%) Grade 2: 4 (19.0%) Grade 3: 1 (4.8%)
Posterior 1/3	Grade 0: 15 (71.4%) Grade 1: 6 (28.6%) Grade 2: 0 (0.0%) Grade 3: 0 (0.0%)
Number of BMLs	
Anterior 1/3	0.7 (0.5)
Middle 1/3	1.2 (0.7)
Posterior 1/3	0.3 (0.5)
% Of lesion that is BML^c^	
Anterior 1/3	1.5 (1.4)
Middle 1/3	2.3 (1.1)
Posterior 1/3	0.7 (1.2)
Cartilage area: any loss^d^	
Anterior 1/3	1.7 (1.1)
Middle 1/3	1.9 (0.7)
Posterior 1/3	0.7 (0.8)
Cartilage area: full thickness loss^d^	
Anterior 1/3	Grade 0: 9 (42.9%) Grade 1: 4 (19.0%) Grade 2: 4 (19.0%) Grade 3: 4 (19.0%)
Middle 1/3	Grade 0: 8 (38.1% Grade 1: 3 (14.3%) Grade 2: 9 (42.9%) Grade 3: 1 (4.8%)
Posterior 1/3	Grade 0: 15 (71.4%) Grade 1: 5 (23.8%) Grade 2: 1 (4.8%) Grade 3: 0 (0.0%)
Medial tibia cartilage thickness (mm)^e^	
Anterior-medial	1.2 (0.5)
Anterior-lateral	1.0 (0.5)
Middle-medial	2.0 (0.6)
Middle-lateral	1.3 (0.4)
Posterior-medial	1.4 (0.3)
Posterior-lateral	1.3 (0.4)

All variables are represented as mean (standard deviation).

∗MOAKS: MRI Osteoarthritis Knee Score. †BML: Bone marrow lesion.

a 11 of the 21 participants had a full-thickness cartilage defect in addition to a BML.

b Scale from 0 to 3; 0: Nil; 1: <33% subregion; 2: 33–66% subregion; 3: >66% subregion.

c Scale from 0 to 3; 0: Nil; 1: <33% of BML; 2: 33–66% of BML; 3: >66% of BML.

d Scale from 0 to 3; 0: Nil; 1: <10% subregion; 2: 10–75% subregion; 3: >75% subregion.

e Cartilage thickness analysis includes 6 subregions, rather than the 3 for the MOAKS.

### 3.2 (Study aim 1) determine the immediate effect of valgus knee brace on medial tibial cartilage contact pressure atop BMLs and full-thickness cartilage defects (where present) during walking

#### 3.2.1 Bone marrow lesions

Of the 21 participants analysed, 15 (71%) exerted cartilage contact pressure atop their BML(s) during unbraced walking, with the remaining 6 (29%) participants having their BML(s) located on non-weight-bearing (i.e., peripheral) regions of the medial tibia (Supplementary Fig. 1). Walking speed was not different between braced and unbraced walking (p = 0.20) (Table 3). The medial tibial cartilage pressure peak magnitude and time-integral across stance atop BMLs was lower by 13% and 17% during braced walking compared to unbraced walking (peak: 1.2 MPa [95%CI −2.0, −0.4], p = 0.008; time-integral: 0.5 MPa s [95%CI -0.9, −0.2], p = 0.009) (Table 3).

**Table 3:** Mean (standard deviation) in walking speed and pressure atop medial tibial BMLs and full-thickness cartilage defects for unbraced and braced walking, with accompanying mean differences and effect sizes (reported as Cohen’s dz), 95% confidence intervals, and p values. Analysis is limited to those participants who exerted pressure atop these structures during walking (n = 15 for BMLs; n = 9 for full-thickness cartilage defects).

	Unbraced walking Mean (SD)	Braced walking Mean (SD)	Mean difference (95%CI) Braced - Unbraced	Effect size (95%CI) Braced - Unbraced	Mean change from Unbraced walking (%)	P Value
Walking speed (m⋅s^−1^)	1.20 (0.14)	1.22 (0.14)	0.02 (−0.01, 0.06)		1.7%	0.20
*Mean cartilage contact pressure atop BML (n = 15)*						
Stance peak magnitude (MPa)	9.1 (3.6)	7.9 (4.0)	−1.2 (−2.0, −0.4)	0.76 (0.49, 0.88)	−13.0%	0.008
Stance time-integral (MPa⋅s)	3.1 (1.5)	2.6 (1.7)	−0.5 (−0.9, −0.2)	0.78 (0.19, 1.35)	−17.1%	0.009
*Mean cartilage contact pressure atop cartilage defect (n = 9)*						
Stance peak magnitude (MPa)	10.5 (4.8)	8.6 (4.9)	−1.9 (−3.3, −0.5)	0.85 (0.65, 0.90)	−18.1%	0.013
Stance time-integral (MPa⋅s)	3.2 (1.9)	2.5 (1.8)	−0.7 (−1.0, −0.4)	1.99 (0.81, 3.13)	−21.9%	<0.001

∗BML: Bone marrow lesion. †Cartilage defect: full-thickness cartilage lesion denuded to subchondral bone. ‡SD: Standard deviation. §95% CI: 95% Confidence Interval. ❡MPa: MegaPascal.

#### 3.2.2 Full-thickness cartilage defects

Of the 21 participants analysed, 11 (52%) had full-thickness cartilage defect(s) on the medial tibia. Of these 11 participants, 9 (82%) exerted pressure atop their cartilage defect(s) during unbraced walking, with the remaining 3 having their defect(s) located on the non-weight-bearing regions of the medial tibia (Supplementary Fig. 1). The medial tibial cartilage contact pressure peak magnitude and time-integral across stance atop full-thickness cartilage defects was 18% and 22% lower during braced walking compared to unbraced walking (peak: 1.9 MPa [95%CI -3.3, −0.5], p = 0.013; time-integral: 0.7 MPa s [95%CI −1.0, −0.4], p < 0.001) (Table 3).

### 3.3 Study aim 2 examine the relationship between individual participant change scores in self-reported knee pain and cartilage pressure atop BMLs/full-thickness cartilage defects

#### 3.3.1 Responder rate descriptive statistics

**Fig. 3:**
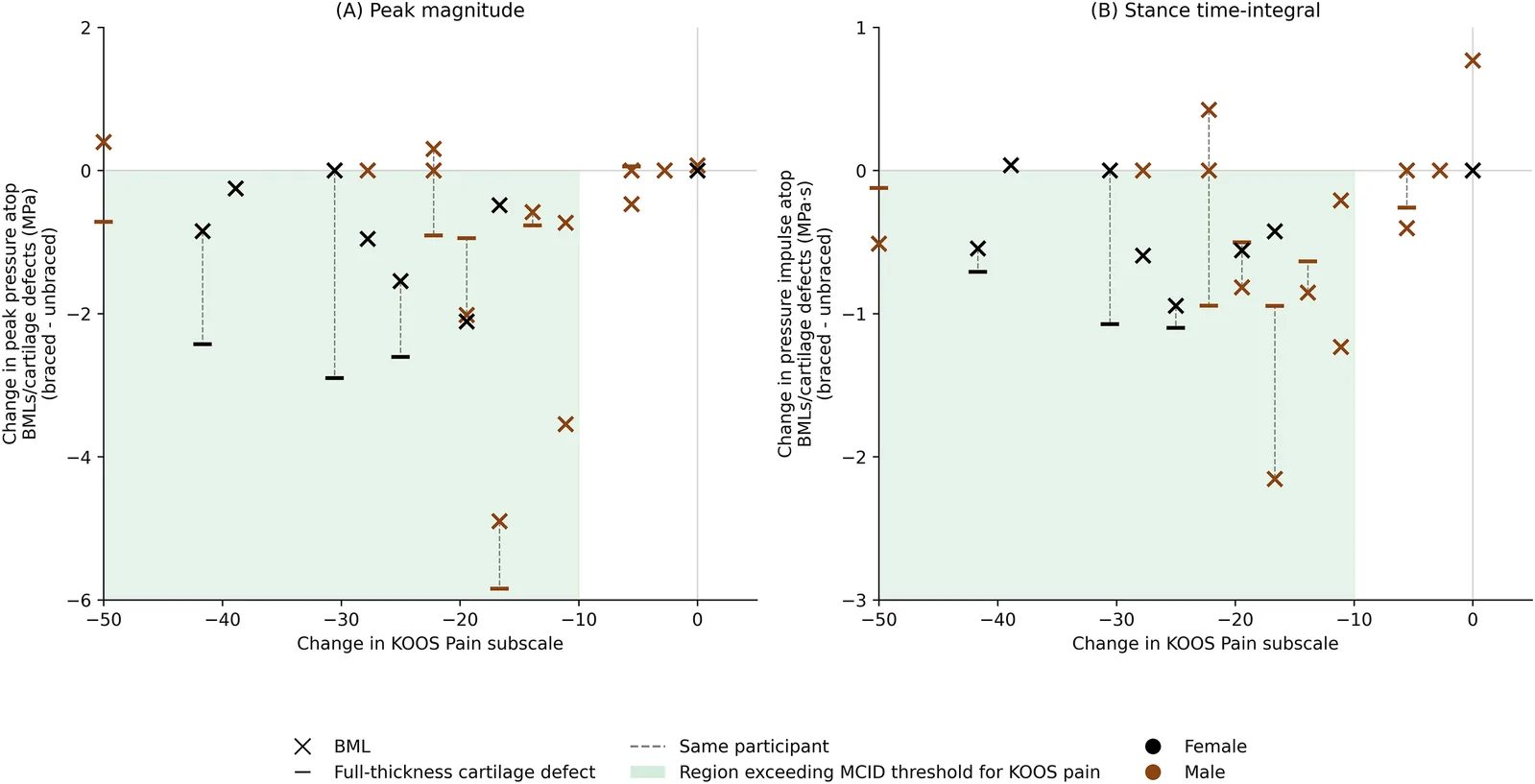
Scatter plots describing individual participant change scores (relative to unbraced walking) for KOOS pain subscale (X coordinate) and cartilage contact pressure peak magnitude (A) and pressure time-integral across stance (B) atop BMLs (cross) and full-thickness cartilage defects (hyphen) (Y coordinate) disaggregated by sex (males: brown; females: black). The dashed line denotes BMLs and full-thickness cartilage defects of the same participant. The light green shade shows the region where KOOS pain change scores exceed the MDIC threshold alongside a reduction in cartilage contact pressure atop the BML or defect.

Of the 21 participants analysed, 16 (76%) reported a reduction in the KOOS pain subscale following the 8-week valgus bracing intervention that exceeded the MCID, and 5 (24%) did not [38] (Table 4, Fig. 3). Of the 16 participants who achieved the MCID in KOOS pain, 14 (88%) reduced cartilage contact pressure atop their full-thickness cartilage defect and/or BML during braced walking. Only one participant (5%) who reduced contact pressure atop their full-thickness cartilage defect and/or BML during braced walking did not reach the MCID in KOOS pain. Inspection of scatter plots reveals no observable effect of sex on data spread (Fig. 3).

**Table 4:** Individual participant change in pain with concurrent change in cartilage pressure atop BMLs, full-thickness cartilage defects during braced walking.

Pain response (n = 16)		No pain response (n = 5)	
Reduced contact pressure atop BML		Reduction in contact pressure atop BML	
Peak (n = 11)	Impulse (n = 12)	Peak (n = 1)	Impulse (n = 1)
Increased contact pressure atop BML		Increased contact pressure atop BML	
Peak (n = 2)	Impulse (n = 1)	Peak (n = 0)	Impulse (n = 1)
No change contact pressure atop BML		No change contact pressure atop BML	
Peak (n = 3)	Impulse (n = 3)	Peak (n = 4)	Impulse (n = 3)
Reduction in contact pressure atop defect		Reduction in contact pressure atop defect	
Peak (n = 8)	Impulse (n = 8)	Peak (n = 0)	Impulse (n = 1)
Increased contact pressure atop defect		Increased contact pressure atop defect	
Peak (n = 0)	Impulse (n = 0)	Peak (n = 0)	Impulse (n = 0)
No change contact pressure atop defect		No change contact pressure atop defect	
Peak (n = 1)	Impulse (n = 1)	Peak (n = 3)	Impulse (n = 2)

∗Only 12 of the 21 participants (57%) had cartilage defect(s) located on the medial tibia in addition to BML(s). Nine of these participants (75%) reported a pain response. †Pain response is a reported a reduction in the KOOS pain subscale following the 8-week valgus bracing intervention that exceeded the MCID value [39]. ‡BML = bone marrow lesion.

#### 3.3.2 Association between reduced pressure atop lesions and pain improvement

Participants who reduced peak pressure atop their full-thickness cartilage defect during braced walking reported greater pain improvement than those who did not (mean difference: 12.9 points [95%CI 0.9, 24.9], p = 0.037) (Table 5). Confidence intervals spanned negative and positive values for peak/stance time-integral pressure atop BMLs and stance pressure time-integral atop cartilage defects, indicating uncertainty in the effect estimate. Participants who reduced peak pressure atop at least one weight-bearing structural pain source reported greater pain improvement than those who did not (mean difference: 13.6 points [95%CI 0.3, 26.9], p = 0.045) (Table 5). Confidence intervals spanned negative and positive values for stance pressure time-integral atop for this combined structural outcome, indicating uncertainty in the effect estimate.

**Table 5:** Comparison of participants who reduced pressure atop weight-bearing BMLs and/or full-thickness cartilage defects during braced walking vs those that did not (others). Mean differences reflect the average difference in KOOS pain change score (points, 0–100 scale) between groups, where positive values indicate greater pain improvement (less pain) in the ‘reduced’ group. Risk differences reflect the difference in the percentage of each group achieving the MCID (i.e., responder rate), where positive values indicate a higher proportion of responders in the ‘reduced’ group.

	n (reduced/others)	KOOS pain change (continuous)			Responder status (≥MCID)		
		Mean difference (95% CI)	Effect size (95% CI)	P Value	Responder rate n (%)	Risk difference (95% CI)	P value
*Reduced pressure atop BML vs. others*							
Peak	12/9	2.7 (−11.4, 16.8)	0.19 (−0.67, 1.06)	0.69	11/12 (92%) vs 5/9 (56%)	36 (−1, 66)	0.12
Impulse	12/9	4.9 (−8.3, 18.0)	0.35 (−0.52, 1.22)	0.44	11/12 (92%) vs 5/9 (56%)	36 (−1, 66)	0.12
*Reduced pressure atop defects vs. others*							
Peak	8/13	12.9 (0.9, 24.9)	1.05 (0.09, 1.97)	0.037	8/8 (100%) vs 8/13 (62%)	38 (0, 64)	0.11
Impulse	9/12	9.7 (−2.7, 22.1)	0.75 (−0.16, 1.63)	0.12	8/9 (89%) vs 8/12 (67%)	22 (−16, 51)	0.34
*Reduced pressure atop BML and/or defect vs. others*							
Peak	15/6	13.6 (0.3, 26.9)	1.10 (0.08, 2.09)	0.045	14/15 (93%) vs 2/6 (33%)	60 (17, 84)	0.011
Impulse	15/6	5.8 (−11.8, 23.4)	0.43 (−0.54, 1.38)	0.46	13/15 (87%) vs 3/6 (50%)	37 (−3, 69)	0.12

*“Others” includes both participants with no measurable contact pressure atop the relevant structure and participants with measurable pressure that did not reduce during braced walking compared to unbraced.

†BML: Bone marrow lesion. †Cartilage defect: full-thickness cartilage lesion denuded to subchondral bone. ‡MCID: Minimal clinically important difference. §95% CI: 95% Confidence Interval. ❡KOOS: Knee injury and Osteoarthritis Outcome Score.

For responder status, confidence intervals spanned negative and positive values for peak and stance pressure-time integral atop BMLs and cartilage defects, indicating uncertainty in the effect estimate (Table 5). Participants who reduced peak pressure atop at least one weight-bearing structural pain source exceeded the KOOS MCID at a higher proportion than those who did not (risk difference: 60 percentage points [95%CI 17, 84], p = 0.011) (Table 5). This was not observed for stance pressure-time integral for this combined structural outcome, with confidence intervals spanning negative and positive values, indicating uncertainty in the effect estimate (Table 5).

#### 3.3.3 Linear relationship between changes in pressure atop BMLs/defects and knee pain

Linear regression revealed no significant association between the KOOS pain change score and change in peak or stance time-integral cartilage contact pressure atop BMLs (Supplementary Table 4). These null findings were robust to non-parametric analyses and were consistent for cartilage contact pressure change atop full-thickness cartilage defects, or when a combined measure of pressure change across structural pain sources, were substituted.

#### 3.3.4 Association between anatomical classification of BML/defect loading and pain improvement

Participants who had a BML located within the middle third of the medial tibial plateau reported greater pain improvement than those who did not (mean difference: 19.4 points [95%CI 12.3, 26.6], p < 0.001) (Supplementary Table 5). Furthermore, participants who had a BML located within the middle tibial third exceeded the KOOS MCID at a higher proportion than those who did not (risk difference: 89 percentage points [95%CI 29, 97], p = 0.008). These associations were not apparent for full-thickness cartilage defects, with the confidence internal spanning large negative and positive effect sizes, indicating uncertainty in the effect estimate. The combined anatomical comparison (BML and/or defect present in the middle third) could not be meaningfully evaluated, as only one participant lacked both lesions in the middle third of the medial tibia.

#### 3.3.5 Ceiling and floor effects

Baseline KOOS pain scores did not differ significantly between responders and non-responders, and baseline score was not significantly correlated with subsequent change, suggesting that ceiling or floor effects did not influence responder classification in this cohort (Supplementary Table 6).

## 4 Discussion

Previous meta-analyses of clinical trial evidence suggests valgus bracing for medial knee OA results in a small-to-moderate decrease in knee pain, with highly variable effect sizes [3]. By identifying patient subgroups who benefit most from valgus bracing, therapy could be targeted to individuals to improve effectiveness. In this study, we hypothesised that valgus bracing would reduce pressure atop BMLs and full-thickness cartilage defects in participants who loaded these structures during walking; and that this biomechanical response would be associated with a clinical response (i.e., KOOS pain improvement). Our findings support our first hypothesis. Using advanced neuro-musculoskeletal models, we found valgus bracing reduced stance time-integral cartilage contact pressure atop BMLs and full-thickness cartilage defects by 17% and 22%, respectively, in the subgroup of participants who exerted pressure atop these structures during unbraced walking. Our second hypothesis was partially supported, with reductions in peak pressure, but not stance time-integral impulse being related to individual participant pain response during braced walking. Collectively, these exploratory findings generate the hypothesis that valgus bracing may impart the most clinical benefit to the subgroup of varus-malaligned medial knee OA patients who mechanically load structural sources of weight-bearing pain during walking.

To our knowledge, this is the first study to examine the spatial relationship between structural sources of weight-bearing pain (BMLs, full-thickness cartilage defects denuded to bone) and a spatially resolved measure of knee cartilage load. In this study, most, but not all, participants exerted pressure atop one or more of their BMLs and full-thickness cartilage defects during unbraced walking, making them ideal candidates to assess our hypothesis regarding the mechanisms of valgus bracing in terms of pain reduction. Notably, BMLs have been shown to be more prevalent in weight-bearing regions in knee OA [40], however they can also occur in non-weight-bearing regions of the medial tibia [41], with aetiology linked to ligament enthesopathy [39] and meniscal pathology [42]. This variability in BML location and origin highlights that not all BMLs are subject to high mechanical load during walking, which may partly explain heterogeneity in pain and treatment response seen in clinical trials where the mechanical linkage of BML in participants was uncontrolled. It is possible that a participant who did not exert pressure atop their BMLs/cartilage defects during walking may do so during other tasks with greater cartilage contact pressure excursion (e.g., squatting or stair climbing/descent, where knee flexion is greater [43]). However, these participants are unlikely to be ideal candidates for valgus bracing, as the brace-induced torque decreases with knee flexion [17,19] and hence its capacity to shift contact pressures away from injurious sites is diminished when the knee is substantially flexed.

In participants who exerted pressure atop their BMLs and full-thickness cartilage defects whilst walking, bracing reduced pressure atop BMLs by a mean of 13% (peak) and 17% (time-integral), and cartilage defects by 18% (peak) and 22% (time-integral). In our previous study of this cohort, we observed an 11% reduction in peak medial tibial cartilage contact pressure during braced compared to unbraced walking [17]. This indicates a non-linear relationship between global changes in cartilage pressure and pressure applied atop BMLs/cartilage defects. Supplementary analysis revealed that compared to unbraced walking, braced walking reduced the area of BML and full-thickness cartilage defects under contact pressure by 15% and 20%, respectively; and increased the distance between cartilage contact centre of pressure and BML/defect location by 17% and 10%, respectively (Supplementary Table 7, Supplementary Fig. 1). Taken together, these findings suggest the observed reductions in cartilage contact pressure atop BMLs/defects were driven by favourable changes in both the magnitude and spatial location of cartilage contact pressure.

Participants who reduced peak pressure atop full-thickness cartilage defects, or a combination of BMLs and/or defects, reported greater pain improvement than those who did not. Furthermore, participants who reduced peak pressure atop BMLs and/or cartilage defects were more likely to exceed the KOOS MCID than those who did not. These relationships were not observed for stance pressure time-integral, which may reflect that mechanonociceptor activation is more closely tied to peak stimulus intensity than to cumulative loading across stance [44]. Moreover, this relationship was not observed for pressure atop BMLs in isolation, suggesting a complex interplay between BMLs and co-occurring cartilage defects that is difficult to disentangle with this current sample.

Our small sample size yielded wide confidence intervals for the regression slopes, precluding any meaningful interpretation of a relationship between the KOOS pain change score and change in cartilage contact pressure atop BMLs or defects. Notably, our study did not have a control arm, which means that a detectable dose-response relationship may be obscured by non-specific brace effects on pain response such as expectation, attention, or placebo. Furthermore, our regression models did not account for brace wear time. However, supplementary analysis of 8-week brace wear-time logbooks revealed that all participants who did not report a pain response to bracing had moderate-to-high wear time; and wear time was negatively correlated with KOOS pain change (r = −0.59, p = 0.006; Supplementary Table 8). This counterintuitive finding may reflect reverse causation (participants reducing brace use as symptoms improved) rather than a true adherence effect, though a control group would be needed to confirm this.

Clinical translation of our study findings requires further research to confirm our exploratory data. Specifically, clinical trial evidence is required to confirm whether valgus bracing improves knee pain more in medial knee OA participants who have structural sources of weight-bearing pain located in regions where the brace can offload, compared to those who do not. If our hypotheses are validated, such evidence could improve clinical decision-making surrounding prescription of valgus bracing, particularly for patients awaiting joint replacement or tibial osteotomy who typically have imaging available. In this context, suitable candidates could be identified based on the presence or absence of structural sources of pain in regions likely to be impacted with valgus bracing, perhaps via *in silico* gait simulations [45]. Interestingly, our finding that anatomical BML presence within the middle third of the tibial plateau alone was associated with pain improvement (Cohen’s d = 1.62), which suggests that, for BMLs specifically, MRI classification alone may be sufficient to identify likely responders (Supplementary Table 5). This was not the case for full-thickness cartilage defects (Cohen’s d = −0.04) or the combined BML/defect measure (not meaningfully assessable), for which subject-specific biomechanical assessment may instead be required. Nonetheless, implementation in routine clinical practice is challenged by the current need for MRI or other imaging to identify BMLs/cartilage defects. A broader application will require investigation of alternative, clinically feasible approaches to identify likely responders. For example, rather than solely relying on structural inclusion criteria as candidates for valgus bracing, patient selection be assisted by proxy measures (e.g., alignment, pain on walking) and/or exclusion criteria (e.g., based on elevated central pain mechanisms [46]).

### 4.1 Limitations

Limitations of this study warrant consideration. This study was not originally powered to detect changes in cartilage contact pressure atop BMLs and full-thickness cartilage defects, thus findings should be considered exploratory, not conclusive. Due to limitations of our MRI sequence (T_1_ VIBE), we performed a 2D spatial analysis between tibial cartilage pressure and the largest cross-sectional area of participant’s BML(s). This method ignores the convoluted shape and volume of the BML and its depth in the subchondral bone, which may influence the magnitude of weight-bearing pain and the spatial associations with the cartilage pressures. Future research using dedicated MRI sequences (e.g., fat-supressed T_2_-weigthed and/or proton density-weighted [47]) and deep learning BML segmentation toolboxes [48] could be used to rapidly analyse 3D contact pressure on BMLs. It is possible that participants with BMLs in non-weight-bearing tibiofemoral regions may still benefit from valgus bracing through other biomechanical mechanisms not examined in this study such as improved arthrokinematics/joint stability [49]. However, such analyses were outside the scope of this study.

Our exploratory pain response data should be interpreted with caution. This study did not include a control or sham-brace group, meaning we cannot determine what proportion of the observed pain response reflects a genuine mechanical effect of valgus bracing. Participant pain response may also have been affected by BMLs and/or defects present at the femur and patella, the mechanics of which were not examined in this study. Pressure data reflect baseline biomechanics only and do not capture potential neuromuscular adaptations in contact pressure atop BMLs/defects over the 8-week intervention.

Our study consisted of 62% males, despite OA affecting females more often than males [50]. While visual inspection did not suggest an apparent effect of sex on the spread of individual participant data (Fig. 3), we did not perform formal statistical comparisons by sex given our low sample size. Future research should examine whether sex-based differences exist in response to valgus bracing and cartilage contact pressure atop BMLs and full-thickness cartilage defects. A single trained researcher performed all MOAKS scoring, and intra- or inter-rater reliability was not formally assessed. Undetected measurement error cannot be excluded as a contributing factor to this result and warrants confirmation with formal reliability assessment in future work. Finally, our findings are only generalisable to those who have varus-malaligned medial knee OA and the specific valgus brace evaluated, however, our analysis methods are completely agnostic and could be applied to any other configuration of patients and orthosis.

## 5 Conclusions

Walking with a valgus brace reduced pressure atop medial tibial BMLs and full-thickness cartilage defects in a subgroup of varus-malaligned medial knee OA participants. This biomechanical response was associated with pain improvement, generating the hypothesis that reduced pressure atop cartilage defects and/or BMLs may underpin the clinical benefit of valgus bracing. Future clinical trial evidence is necessary to confirm these exploratory findings and determine the most appropriate patients for valgus bracing intervention.

## Contributions

SCS, MH, AE, and DJS conceived the idea for the paper. SCS and more of the authors (MH) acquired the data. SCS, AE, MH, and WMO performed data analysis. All authors interpreted the data. SCS wrote the first draft of the manuscript. All authors revised the manuscript for intellectual content and approved the final version for submission. MH obtained the funding for the project from which the data used in this manuscript was derived.

## Role of funding sources

The institution of one or more of the authors (MH) has received, during the study period, funding from the Australian National Health and Medical Research Council (Investigator grant #1172928), the Australian Department of Industry, Innovation and Science (Innovations Connections grant #ICG000857), and Össur Orthopaedic Solutions. The institution of one or more of the authors (AE) has received, during the study period, funding from the European Union’s Horizon 2020 research and innovation programme under the Marie Skłodowska-Curie scholarship (#101108335) and Finnish Cultural Foundation (#00230091). The institution of one or more of the authors (LD) has received, during the study period, funding from the Australian National Health and Medical Research Council (Investigator grant (#2017012). The institution of one or more of the authors (RKK) has received, during the study period, funding from The Research Council of Finland (#363459) and Novo Nordisk foundation (#NNF21OC0065373). The institution of one or more of the authors (DJH) has received, during the study period, funding from the Australian National Health and Medical Research Council (Investigator grant (#1194737). The funders had no role in the design and conduct of the study, the collection, management, data analysis, and interpretation, or the preparation, review, or approval of the manuscript or the decision to submit for publication.

## Conflict of interest

DJS is the Director of Ontic Ortho Solutions Pty Ltd. DJH provides consulting advice on scientific advisory boards for Haleon, Naia Health, Novartis, Lilly, Tissuegene, Doron, Sanofi, Formation Bio, Enlivex; is Editor in Chief, Osteoarthritis and Cartilage; is section editor for osteoarthritis, UpToDate; and is a Board Member of Osteoarthritis Research Society International.
