# Supplementary material for: Valgus bracing can relocate cartilage contact pressure away from bone marrow lesions and full-thickness cartilage defects in varus-malaligned medial knee osteoarthritis
Source: Osteoarthr Cartil Open. 2026 Aug 28;8(4):100871. doi: 10.1016/j.ocarto.2026.100871 (PMC13573189; doi:10.1016/j.ocarto.2026.100871)
Supplement: Supplementary file 1 [file mmc1.pdf]

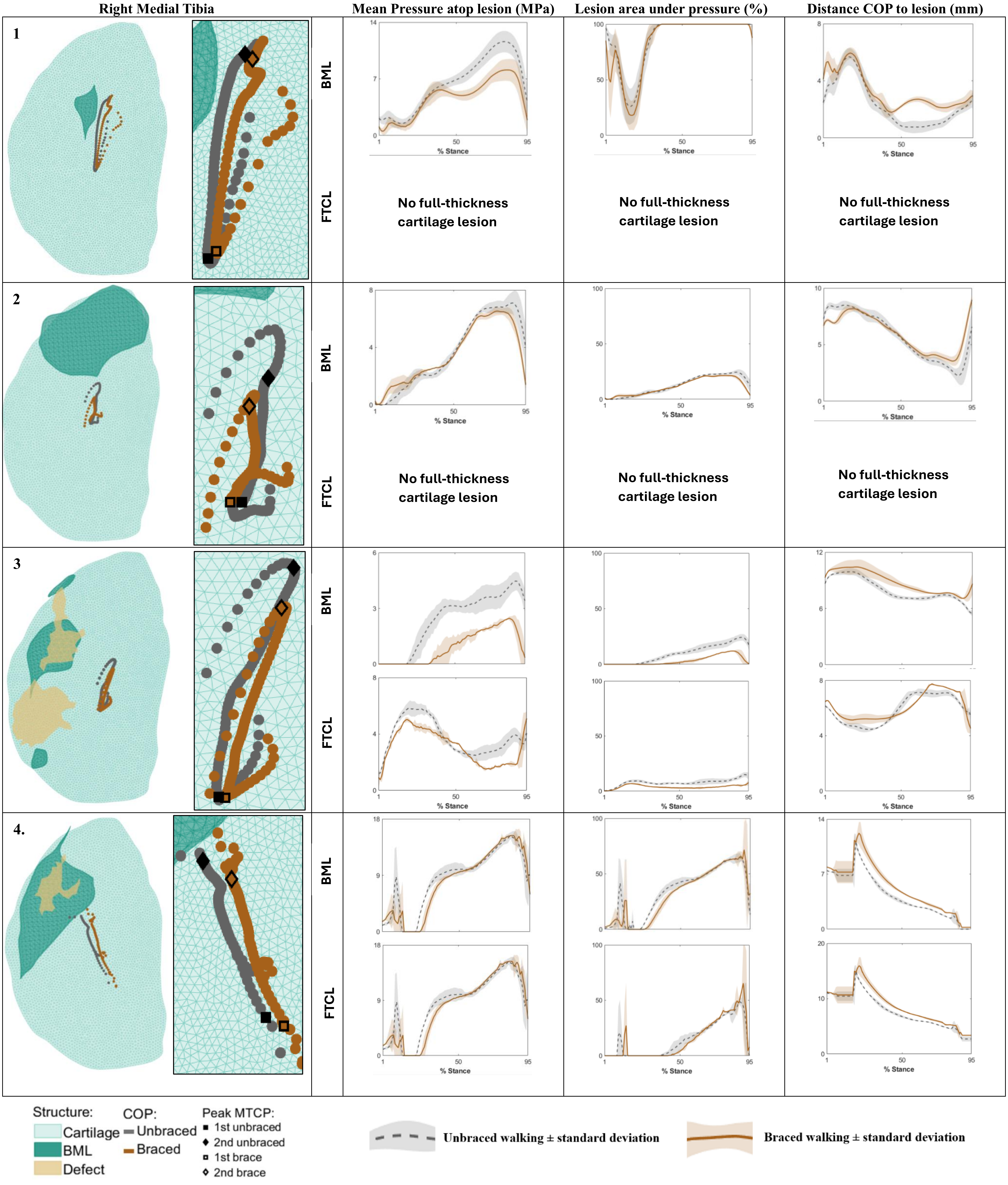
Right Medial Tibia
Mean Pressure atop lesion (MPa)
Lesion area under pressure (%)
Distance COP to lesion (mm)
1
BML
FTCL
% Stance
No full-thickness cartilage lesion
No full-thickness cartilage lesion
No full-thickness cartilage lesion
2
BML
FTCL
% Stance
No full-thickness cartilage lesion
No full-thickness cartilage lesion
No full-thickness cartilage lesion
3
BML
FTCL
% Stance
4.
BML
FTCL
% Stance
Structure:
Cartilage
BML
Defect
COP:
Unbraced
Braced
Peak MTCP:
1st unbraced
2nd unbraced
1st brace
2nd brace
Unbraced walking ± standard deviation
Braced walking ± standard deviation

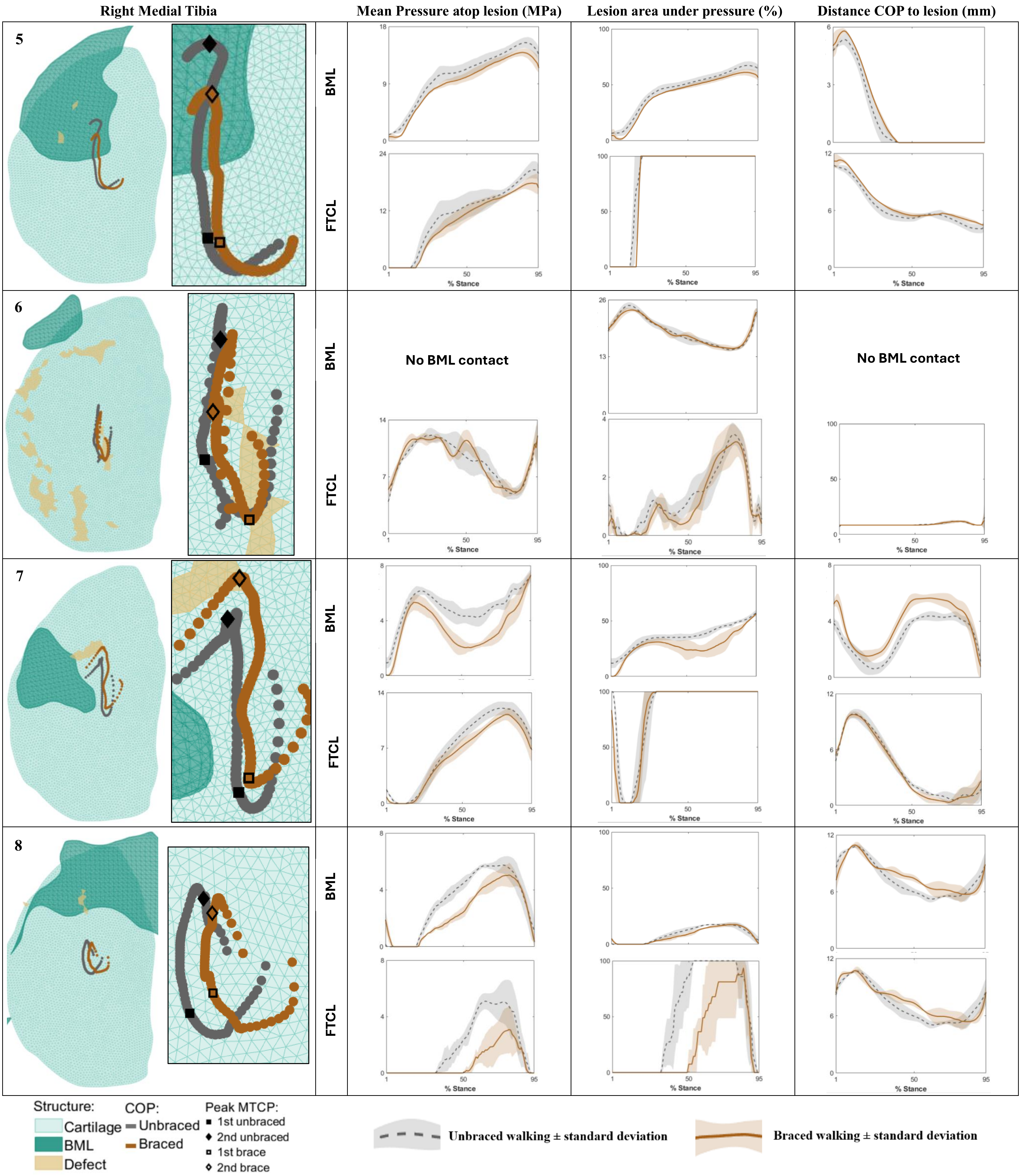

Right Medial Tibia
Mean Pressure atop lesion (MPa)
Lesion area under pressure (%)
Distance COP to lesion (mm)
5
6
7
8
BML
FTCL
No BML contact
No BML contact
% Stance
Structure:
Cartilage
BML
Defect
COP:
Unbraced
Braced
Peak MTCP:
1st unbraced
2nd unbraced
1st brace
2nd brace
Unbraced walking ± standard deviation
Braced walking ± standard deviation

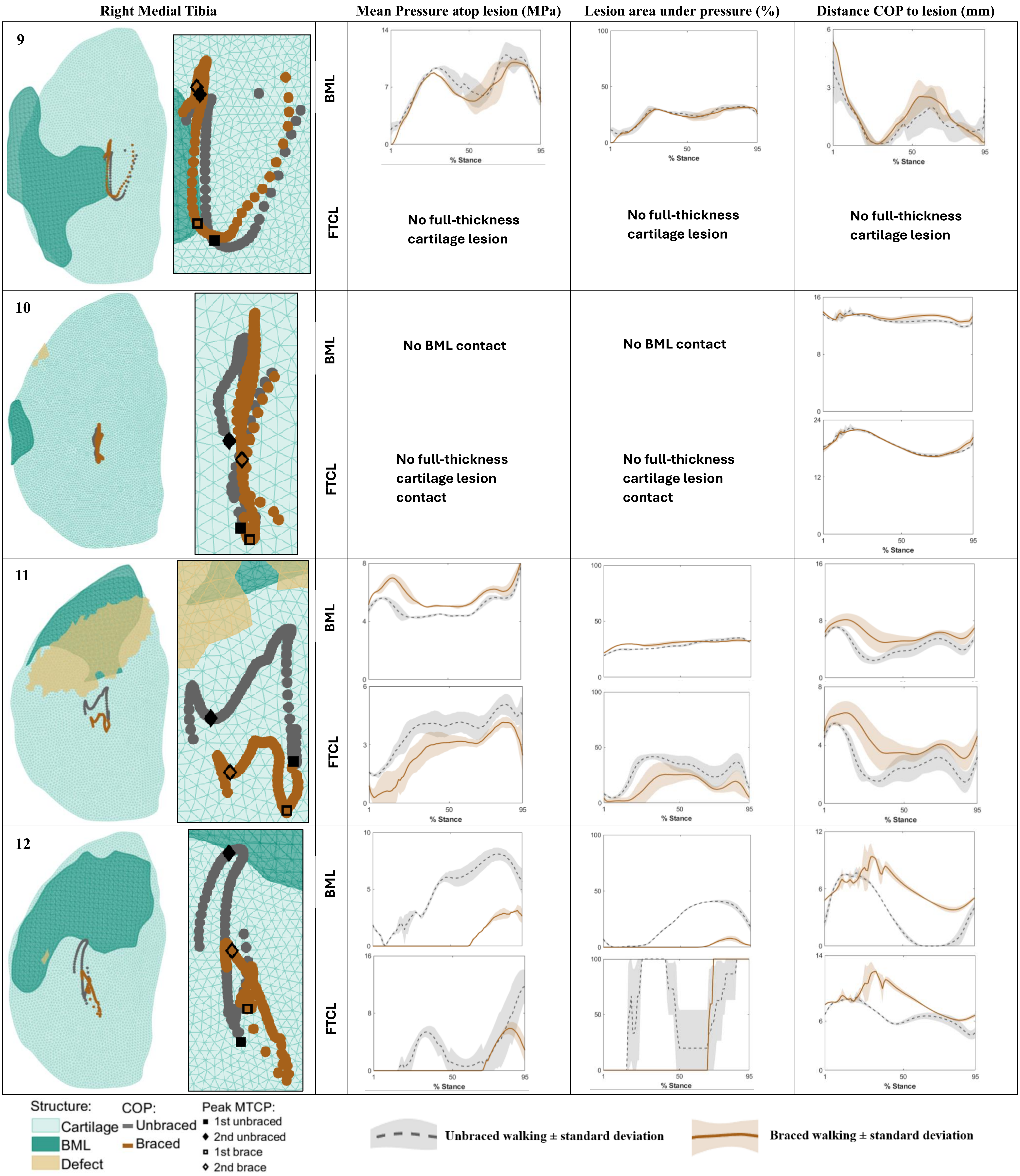

Right Medial Tibia
Mean Pressure atop lesion (MPa)
Lesion area under pressure (%)
Distance COP to lesion (mm)
9
BML
FTCL
No full-thickness cartilage lesion
No full-thickness cartilage lesion
No full-thickness cartilage lesion
10
BML
FTCL
No BML contact
No BML contact
No full-thickness cartilage lesion contact
No full-thickness cartilage lesion contact
11
BML
FTCL
12
BML
FTCL
% Stance
Structure:
Cartilage
BML
Defect
COP:
Unbraced
Braced
Peak MTCP:
1st unbraced
2nd unbraced
1st brace
2nd brace
Unbraced walking ± standard deviation
Braced walking ± standard deviation

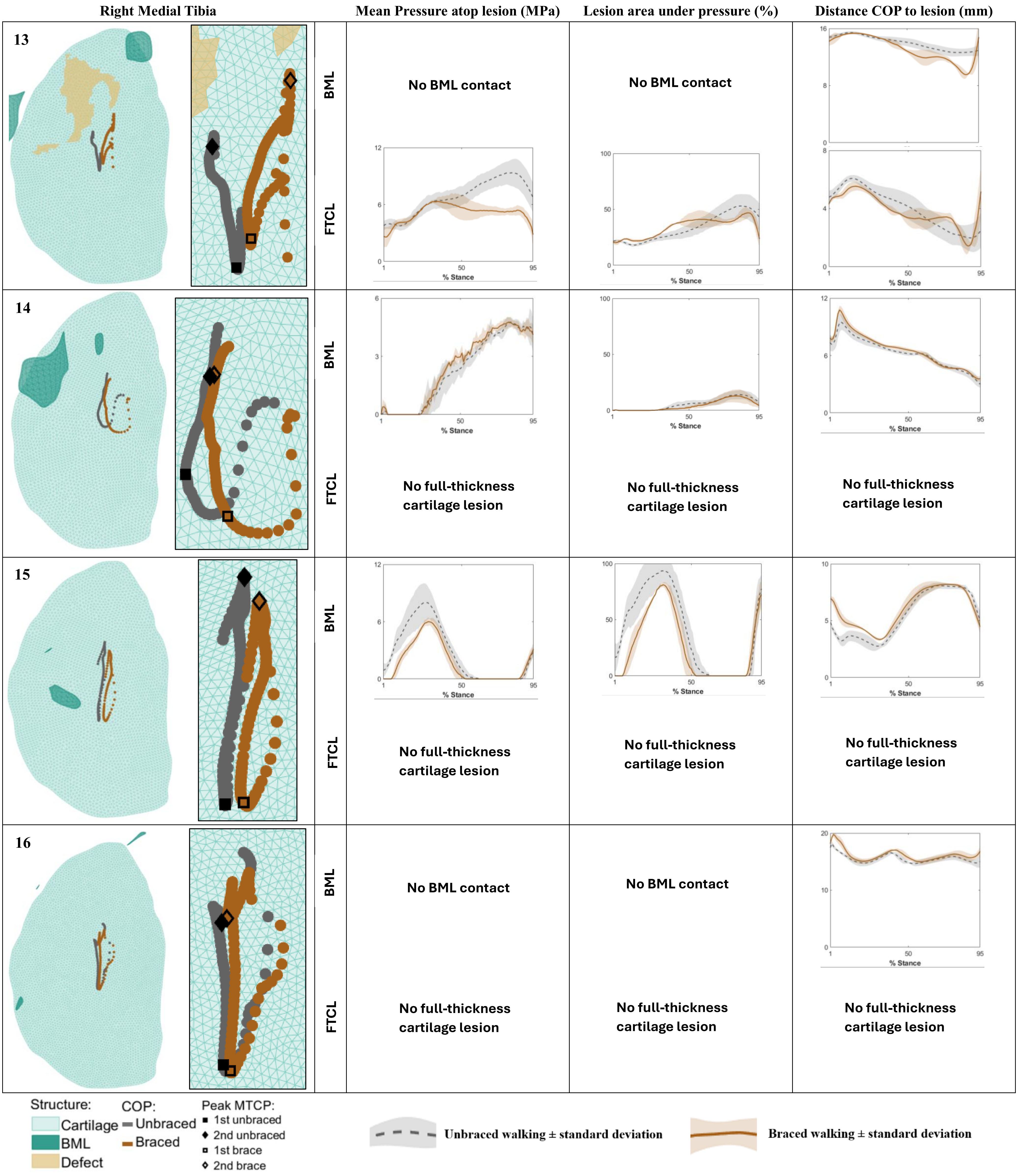
Right Medial Tibia
Mean Pressure atop lesion (MPa)
Lesion area under pressure (%)
Distance COP to lesion (mm)
13
14
15
16
BML
FTCL
No BML contact
No full-thickness cartilage lesion
% Stance
Structure:
Cartilage
BML
Defect
COP:
Unbraced
Braced
Peak MTCP:
1st unbraced
2nd unbraced
1st brace
2nd brace
Unbraced walking ± standard deviation
Braced walking ± standard deviation

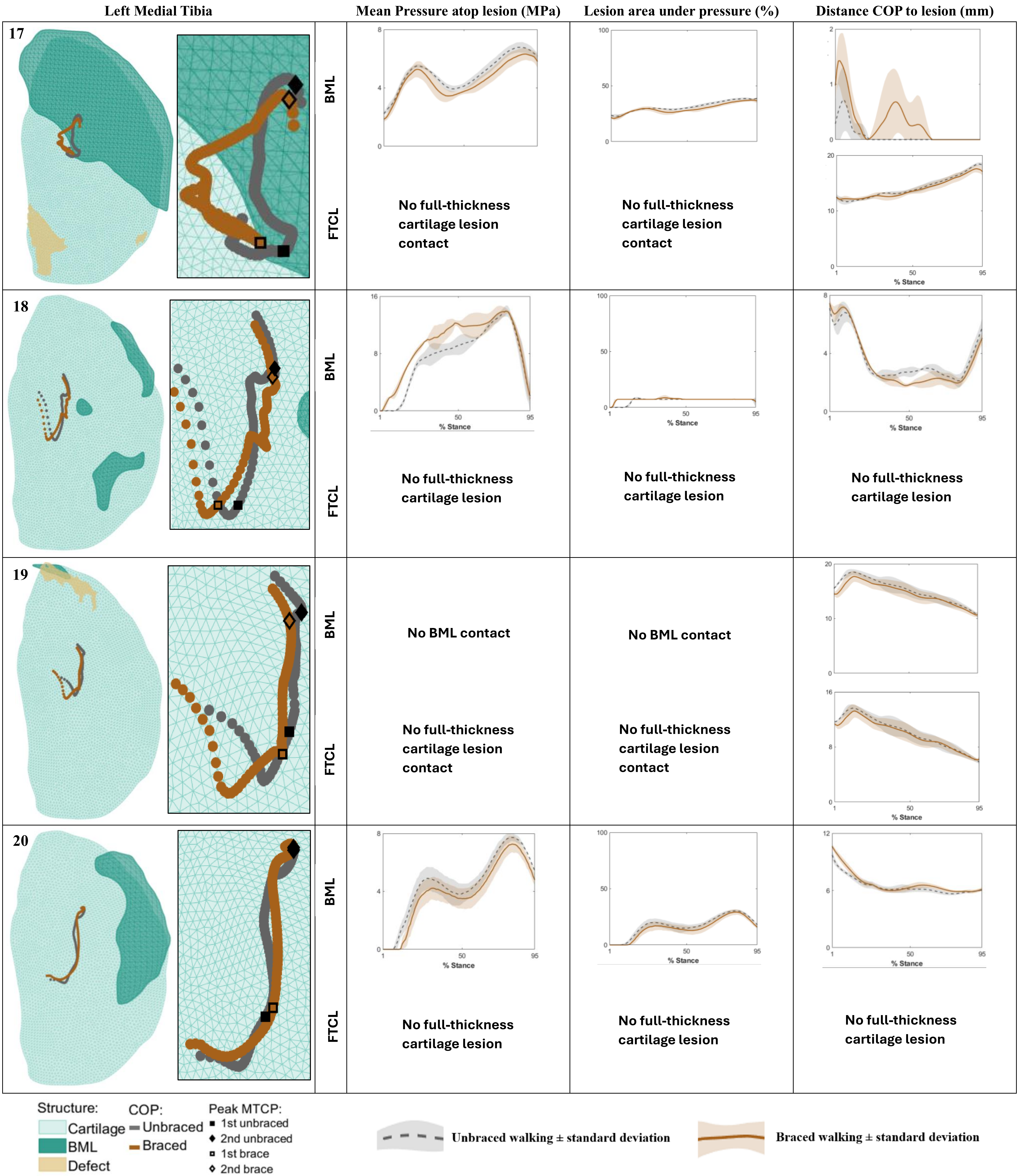

Left Medial Tibia
Mean Pressure atop lesion (MPa)
Lesion area under pressure (%)
Distance COP to lesion (mm)
17
BML
FTCL
No full-thickness cartilage lesion contact
No full-thickness cartilage lesion contact
% Stance
18
BML
FTCL
% Stance
No full-thickness cartilage lesion
No full-thickness cartilage lesion
No full-thickness cartilage lesion
19
BML
FTCL
No BML contact
No BML contact
No full-thickness cartilage lesion contact
No full-thickness cartilage lesion contact
% Stance
20
BML
FTCL
% Stance
No full-thickness cartilage lesion
No full-thickness cartilage lesion
No full-thickness cartilage lesion
Structure:
Cartilage
BML
Defect
COP:
Unbraced
Braced
Peak MTCP:
1st unbraced
2nd unbraced
1st brace
2nd brace
Unbraced walking ± standard deviation
Braced walking ± standard deviation

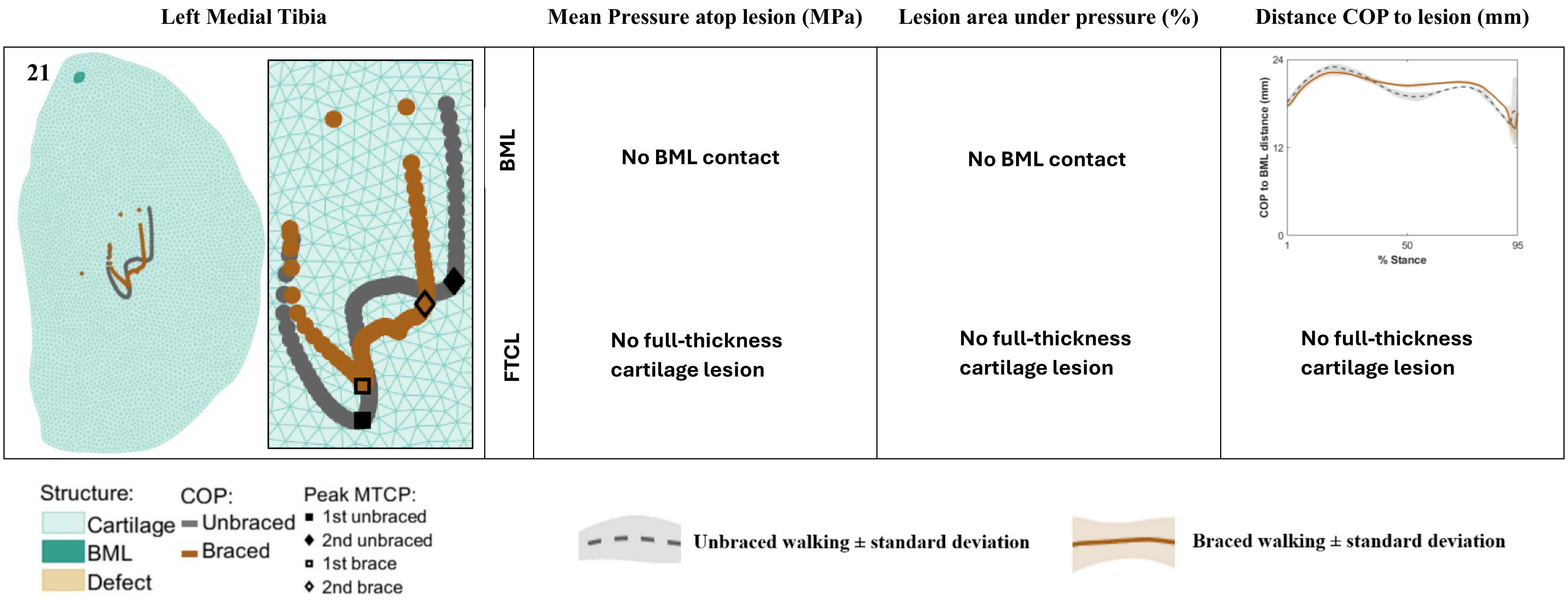

*BML: Bone marrow lesion. †FTCL: full-thickness cartilage lesion denuded to subchondral bone. ‡ MPa: MegaPascal.§COP: cartilage contact centre of pressure. ¶MTCP: Mean tibiofemoral cartilage contact pressure

Supplementary Fig 1. Spatial maps of individual participant medial tibial contact centre of pressure from 0 to 95% of stance during unbraced (grey line) and braced (brown line) walking; alongside spatial location of medial tibial cartilage (light blue), bone marrow lesions (green), and full-thickness cartilage defects denuded to bone where present (tan). Square and diamond symbols denote the spatial location of the cartilage centre of pressure at the 1st and 2nd peak medial tibiofemoral cartilage contact pressure, respectively. Plots show the time-varying mean ± standard deviation pressure atop lesion (MPa), area lesion under cartilage contact pressure (as a % of medial tibial cartilage area), and distance from cartilage contact centre of pressure to lesion (mm) during unbraced (grey line ± shaded standard deviation) and braced (brown line ± shaded standard deviation) walking.
