# Supplementary material for: Valgus bracing can relocate cartilage contact pressure away from bone marrow lesions and full-thickness cartilage defects in varus-malaligned medial knee osteoarthritis
Source: Osteoarthr Cartil Open. 2026 Aug 28;8(4):100871. doi: 10.1016/j.ocarto.2026.100871 (PMC13573189; doi:10.1016/j.ocarto.2026.100871)
Supplement: Supplementary file 2 [file mmc2.docx]

**Supplementary Table 1.** STROBE checklist.

| **Section/Topic** | **Item #** | **Recommendation** | **Reported on page/section** |
| --- | --- | --- | --- |
| Title and abstract | 1a | Indicate the study's design with a commonly used term in the title or the abstract | Abstract, Design (“within-participant longitudinal study”). |
|  | 1b | Provide in the abstract an informative and balanced summary of what was done and what was found | Abstract (structured: Objective, Design, Methods, Results, Conclusions) |
| Introduction |  |  |  |
| Background/rationale | 2 | Explain the scientific background and rationale for the investigation being reported | Introduction, para 1–3 |
| Objectives | 3 | State specific objectives, including any prespecified hypotheses | Introduction, final paragraph |
| Methods |  |  |  |
| Study design | 4 | Present key elements of study design early in the paper | Methods, opening paragraph |
| Setting | 5 | Describe the setting, locations, and relevant dates, including periods of recruitment, exposure, follow-up, and data collection | Methods – Participants (recruitment dates/location); Procedures (testing location); magnetic resonance imaging (imaging location) |
| Participants | 6a | Cohort study—Give the eligibility criteria, and the sources and methods of selection of participants. Describe methods of follow-up | Methods – Participants (inclusion/exclusion criteria, secondary-analysis eligibility criteria); procedures (follow-up testing at 8 weeks) |
|  | 6b | Cohort study—For matched studies, give matching criteria and number of exposed and unexposed | Not applicable (unmatched, single-arm design) |
| Variables | 7 | Clearly define all outcomes, exposures, predictors, potential confounders, and effect modifiers. Give diagnostic criteria, if applicable | Methods – Data acquisition (KOOS pain, biomechanical data); data analysis (cartilage contact pressure, BML/defect definitions per MOAKS) |
| Data sources/measurement | 8* | For each variable of interest, give sources of data and details of methods of assessment (measurement). Describe comparability of assessment methods if there is more than one group | Methods – data acquisition (Biomechanical data, Magnetic resonance imaging, self-reported knee pain subsections); data analysis (BML/defect segmentation, pressure calculation) |
| Bias | 9 | Describe any efforts to address potential sources of bias | Partially addressed: single-scorer MOAKS assessment and 2D BML segmentation acknowledged in Discussion/Limitations rather than a dedicated Methods statement. |
| Study size | 10 | Explain how the study size was arrived at | Methods – Participants (“a formal a priori sample size/power calculation was not performed…”) |
| Quantitative variables | 11 | Explain how quantitative variables were handled in the analyses. If applicable, describe which groupings were chosen and why | Statistical analysis for study aim 2 (KOOS transformation to change scores; dichotomisation of pressure change at zero; middle-third MOAKS grouping rationale) |
| Statistical methods | 12a | Describe all statistical methods, including those used to control for confounding | Statistical analysis (overview); Statistical analysis for study aim 1 and 2 (paired t-tests, Welch’s t-tests, Fisher’s exact test, linear regression). |
|  | 12b | Describe any methods used to examine subgroups and interactions | Statistical analysis for study aim 2 (BML vs. defect vs. combined comparisons; anatomical vs. biomechanical classification). Sex comparison was descriptive/visual only (Figure 3), not a formal subgroup analysis – stated as a limitation. |
|  | 12c | Explain how missing data were addressed | Participant exclusions are explained (Results, Cohort description) and one participant’s missing wear-time data is footnoted (Supplementary Table). |
|  | 12d | Cohort study—If applicable, explain how loss to follow-up was addressed | No loss to follow-up between baseline and 8-week assessment is reported; all 21 analysed participants completed both timepoints (Figure 1). |
|  | 12e | Describe any sensitivity analyses | Statistical analysis for study aim 2 (Spearman’s rank correlation as regression sensitivity check); Discussion (sensitivity check excluding non-adherent participant in wear-time correlation) |
| Results |  |  |  |
| Participants | 13a* | Report numbers of individuals at each stage of study—eg numbers potentially eligible, examined for eligibility, confirmed eligible, included in the study, completing follow-up, and analysed | Figure 1 (flow diagram); Results, Cohort description (21 of 28 included, reasons for exclusion of 7) |
|  | 13b | Give reasons for non-participation at each stage | Results, Cohort description (MRI artifacts n=3; no medial tibial BML n=4) |
|  | 13c | Consider use of a flow diagram | Figure 1 |
| Descriptive data | 14a* | Give characteristics of study participants (eg demographic, clinical, social) and information on exposures and potential confounders | Table 1 (demographics); Table 2 (MOAKS/cartilage characteristics); Results, Cohort description |
|  | 14b | Indicate number of participants with missing data for each variable of interest | Partially reported: pressure variables (15/21 exerted pressure atop BML, 9/11 atop defect – Results, Study aim 1) and wear time (1 participant missing – Supplementary Table footnote). |
|  | 14c | Cohort study—Summarise follow-up time (eg, average and total amount) | Fixed 8-week follow-up for all participants by design (Methods). |
| Outcome data | 15* | Cohort study—Report numbers of outcome events or summary measures over time | Results, Study aim 2 – Responder rate descriptive statistics (16/21 achieved MCID, 5/21 did not; Table 4, Figure 3) |
| Main results | 16a | Give unadjusted estimates and, if applicable, confounder-adjusted estimates and their precision (eg, 95% confidence interval). Make clear which confounders were adjusted for and why they were included | Tables 3–5, Supplementary Tables 4–6, 8 (all estimates unadjusted, with 95% CIs throughout). No confounder adjustment was performed due to small sample size. |
|  | 16b | Report category boundaries when continuous variables were categorized | Statistical analysis for study aim 2 (pressure change dichotomised at zero i.e. reduced vs. not reduced; KOOS pain dichotomised at the MCID threshold of 10 points) |
|  | 16c | If relevant, consider translating estimates of relative risk into absolute risk for a meaningful time period | Not applicable – risk differences (absolute) reported directly throughout (Table 5, Supplementary Tables 6, 8) rather than relative risk |
| Other analyses | 17 | Report other analyses done—eg analyses of subgroups and interactions, and sensitivity analyses | Results, Study aim 2 (ceiling/floor effect analysis; anatomical MOAKS classification analysis; linear regression with sensitivity analyses); Discussion (wear-time/adherence analysis) |
| Discussion |  |  |  |
| Key results | 18 | Summarise key results with reference to study objectives | Discussion, opening paragraphs (restates both hypotheses and whether supported) |
| Limitations | 19 | Discuss limitations of the study, taking into account sources of potential bias or imprecision. Discuss both direction and magnitude of any potential bias | Discussion – Limitations subsection (power, 2D BML segmentation, absence of control group, femur/patella BMLs not examined, baseline-only pressure data, sex imbalance, MOAKS scorer reliability, generalisability) |
| Interpretation | 20 | Give a cautious overall interpretation of results considering objectives, limitations, multiplicity of analyses, results from similar studies, and other relevant evidence | Discussion (throughout; explicit hypothesis-generating/exploratory framing); Conclusions |
| Generalisability | 21 | Discuss the generalisability (external validity) of the study results | Discussion – Limitations, final sentence (generalisable only to varus-malaligned medial knee OA and the specific brace evaluated) |
| Other information |  |  |  |
| Funding | 22 | Give the source of funding and the role of the funders for the present study and, if applicable, for the original study on which the present article is based | ROLE OF FUNDING SOURCES section |

**Supplementary Table 2.** Summary of MRI Scan Protocol.

Fat-suppressed, isotropic 3D T1-weighted VIBE (Volume Interpolated Breath-hold Examination) gradient-echo sequence, acquired in sagittal plane.

| **Scanner** | Siemens Prisma_fit, 3T |
| --- | --- |
| **Software Version** | Syngo MR E11 |
| **Study Date** | 2019 |
| **Body Part Examined** | Knee |
| **Series Description** | t1_vibe_we_sag_iso_p2 |
| **Patient Position** | Feet-First Supine (FFS) |

## Sequence

| **Sequence Type** | GR (Gradient Echo), Spoiled (SP) |
| --- | --- |
| **Sequence Name** | *fl3d1 (3D FLASH-type — VIBE) |
| **Acquisition Type** | 3D |
| **Scan Options** | SAT1, WE (Water Excitation — fat suppression) |
| **Echo Train Length** | 1 |
| **Number of Averages** | 1 |

## Timing / Contrast

| **Repetition Time (TR)** | 11.7 ms |
| --- | --- |
| **Echo Time (TE)** | 5.26 ms |
| **Flip Angle** | 10° |
| **Inversion Time (TI)** | Not applicable (no IR preparation) |

## Geometry / Resolution

| **Slice Thickness** | 0.8 mm |
| --- | --- |
| **Pixel Spacing** | 0.781 × 0.781 mm |
| **Matrix (Rows × Columns)** | 576 × 288 |
| **Derived Field of View (FOV)** | ~450 × 225 mm (row × column) |
| **Percent Phase FOV** | 50% |
| **Percent Sampling** | 100% |
| **Number of Slices/Instances** | 208 |
| **Pixel Bandwidth** | 190 Hz/px |

**Supplementary Table 3.** Summary of parameters used during musculoskeletal model calibration and medial tibial cartilage contact pressure simulations

| **Cartilage model parameters** |  |
| --- | --- |
| Young’s modulus | 20MPa |
| Poisson ratio | 0.3 |
| **Model muscle maximal isometric force adjustment^a^** | Targets: addbrev', 'addlong', 'addmag', 'bflh','bfsh', 'edl', 'ehl', 'fdl', 'fhl', 'gaslat', 'gasmed', 'glmax1', 'glmed', 'glmin', 'grac', 'iliacus', 'perbrev', 'perlong', 'psoas', 'recfem', 'sart', 'semimem', 'semiten', 'soleus', 'tfl', 'tibant', 'tibpost', 'vasint', 'vaslat', 'vasmed’ |
| **Optimization of model musculotendon parameters^b^** | Targets: addbrev, addlong, addmagDist, addmagIsch, addmagMid, addmagProx, bflh, semimem, sart, bfsh, gaslat, gasmed, glmax1, glmax2, glmax3, glmed1, glmed2, glmed3, glmin1, glmin2, glmin3, grac, recfem, tfl, iliacus, psoas, perbrev, perlong, tibant, pertert, soleus, edl, ehl, fdl, fhl, vasintr, vaslat, vasmed. |
| **CEINMS objective functions for model calibration** |  |
| torqueErrorNormalised | Dofs: hip_flex, knee_flex, ankle_flex  Targets: all  Weight: 1  Exponent: 2 |
| maxSumPeak | Targets: addbrev, addlong, addmagDist, addmagIsch, addmagMid, addmagProx, bflh, semimem, sart, bfsh, gaslat, gasmed, glmax1, glmax2, glmax3, glmed1, glmed2, glmed3, glmin1, glmin2, glmin3, grac, recfem, tfl, iliacus, psoas, perbrev, perlong, tibant, pertert, soleus, edl, ehl, fdl, fhl, vasintr, vaslat, vasmed.  TargetsType: muscleForce  Weight: 5  Exponent: 1 |
| normalisedFibreLength | Weight: 20000  Exponent: 1  Range: 0.8 1.2 |
| **CEINMS model muscle-tendon-unit calibration parameters** |  |
| shapeFactor | Range: [-2.999 -0.001] |
| tendonSlackLength | Range: [0.85 1.15] |
| optimalFibreLength | Range: [0.85 1.15] |
| strengthCoefficient | Range: [0.5 2] |
| **Concurrent Optimization of Muscle Activations and Kinematics (COMAK) settings** | Lowpass filter: 6Hz  Activation exponent: 2  Contact energy weight: 1  Non-muscle actuator weight: 0.5  EMG gamma weight: 200 (all muscles) |

**^a^**As per Handsfield GG, Meyer CH, Hart JM, Abel MF, Blemker SS. Relationships of 35 lower limb muscles to height and body mass quantified using MRI. *J Biomech*. 2014;47:631-638.

**^b^**As per Modenese L, Ceseracciu E, Reggiani M, Lloyd DG. Estimation of musculotendon parameters for scaled and subject specific musculoskeletal models using an optimization technique. *J Biomech.* 2016;49:141-148.

| **Supplementary Table 4.** Linear regression modelling the association between change in KOOS pain and change in cartilage contact pressure atop BMLs, with a non-parametric sensitivity analysis (Spearman's ρ), and substitution of cartilage defect pressure change and a combined structural pressure measure (largest pressure reduction across BMLs and defects). Analysis \| n \| β (95% CI) \| R² \| p; *Primary — BML* \| \| \| \|; Peak pressure ~ KOOS change \| 15 \| 0.025 (-0.038, 0.087) \| 0.05 \| 0.41; Time-integral ~ KOOS change \| 15 \| -0.003 (-0.032, 0.027) \| <0.01 \| 0.86; *Sensitivity — BML* \| \| \| \|; Spearman's ρ (peak) \| 15 \| ρ=0.14 \| \| 0.62; Spearman's ρ (time-integral) \| 15 \| ρ=-0.04 \| \| 0.90; *Sensitivity — substituting defect / combined* \| \| \| \|; Defect peak ~ KOOS change \| 9 \| 0.014 (-0.118, 0.146) \| 0.01 \| 0.81; Defect time-integral ~ KOOS change \| 9 \| 0.009 (-0.016, 0.034) \| 0.10 \| 0.41; Combined peak ~ KOOS change \| 17 \| -0.005 (-0.071, 0.060) \| <0.01 \| 0.87; Combined time-integral ~ KOOS change \| 17 \| -0.006 (-0.032, 0.020) \| 0.02 \| 0.63 *Combined = the largest pressure reduction across whichever structural pain source(s) (BML and/or defect) a participant had. †β = unstandardised regression coefficient. **Supplementary Table 5.** Comparison of KOOS pain change between participants with and without a weight-bearing bone marrow lesion (BML) and/or full-thickness cartilage defect located in the middle third of the medial tibial plateau. Mean differences reflect the difference in KOOS pain change score (points, 0–100 scale) between groups, where positive values indicate greater pain improvement in the group with the lesion present. Risk differences reflect the difference in the percentage of each group achieving the MCID (i.e., responder rate), where positive values indicate a higher proportion of responders in the group with the lesion. \| \| KOOS Pain Change (Continuous) \| \| \| Responder Status (≥MCID) \| \|; Anatomical classification (MOAKS, middle third) \| n (with/without) \| Mean difference (95% CI) \| Effect size (95% CI) \| P value \| Responder rate n (%) \| Risk difference (95% CI) \| P value; BML \| 18/3 \| 19.4 (12.3, 26.6) \| 1.62 (0.27, 2.92) \| <0.001 \| 16/18 (89%) vs 0/3 (0%) \| 89 (29, 97) \| 0.008; Defect \| 13/8 \| -0.6 (-14.8, 13.7) \| -0.04 (-0.92, 0.84) \| 0.933 \| 10/13 (77%) vs 6/8 (75%) \| 2 (-31, 39) \| 1.000; Combined (BML and/or defect) \| 20/1 \| not meaningfully testable* \| \| \| \| \| ***Only 1 participant lacked both a middle-third BML and middle-third defect, precluding analysis. †BML: Bone marrow lesion. Cartilage defect: full-thickness cartilage lesion denuded to subchondral bone. ‡MCID: Minimal clinically important difference. §95% CI: 95% Confidence Interval. ❡KOOS: Knee Osteoarthritis Outcome Score. MOAKS: MRI Osteoarthritis Knee Score. **Supplementary Table 6.** Assessment of ceiling and floor effects on KOOS pain responder classification (n=21). Analysis \| Statistic \| Value; *Participants scoring at scale extremes (0 or 100 points)* \| \|; Baseline \| n (%) \| 0/21 (0%); Follow-up (8 weeks) \| n (%) \| 0/21 (0%); *Baseline KOOS pain by responder status* \| \|; Responders (≥MCID) \| Mean (SD), n=16 \| 50.0 (13.5); Non-responders \| Mean (SD), n=5 \| 50.6 (15.0); Responders − Non-responders \| Mean difference (95%CI) \| -0.6 (-18.8, 17.7); Between-group comparison \| Welch's t-test, p \| 0.94; *Correlation between baseline score and subsequent change* \| \|; Baseline KOOS pain vs. change in KOOS pain \| Pearson's r \| -0.35; \| p \| 0.12 *KOOS pain baseline range 27.8–66.7; follow-up range 27.8–91.7. No participant scored at the scale extremes at either timepoint. **Supplemental Table** **7.** Mean (standard deviation) in walking speed and spatial variables relating to medial tibial cartilage pressure atop medial tibial BMLs and full-thickness cartilage defects for unbraced and braced walking, with accompanying mean differences and 95% confidence intervals. Analysis is limited to those participants who exerted pressure atop these structures during walking (n=15 for BMLs; n=9 for full-thickness cartilage defects). | | | | |
| --- | --- | --- | --- | --- |
|  | Unbraced walking  Mean (SD) | Braced walking  Mean (SD) | Mean difference (95%CI)  Braced - Unbraced | Mean change from Unbraced walking (%) |
| Walking speed (m·s^-1^) | 1.20 (0.14) | 1.22 (0.14) | 0.02 (-0.01, 0.06) | 1.7% |
| *Amount of lesion under cartilage contact pressure (% area)* |  |  |  |  |
| BML stance mean (n=15) | 27.2 (20.9) | 23.1 (21.1) | -4.1 (-7.3, -1.0) | -15.1% |
| Cartilage defect stance mean (n=9) | 40.2 (28.9) | 32.3 (28.9) | -7.9 (-16.0, 0.2) | -19.7% |
| *Mean distance between cartilage contact COP and lesion (mm)* |  |  |  |  |
| BML stance mean (n=15) | 4.2 (2.4) | 4.9 (2.5) | 0.7 (0.3, 1.1) | 16.7% |
| Cartilage defect stance mean (n=9) | 5.1 (2.2) | 5.6 (2.5) | 0.5 (0.0, 1.1) | 9.8% |

*BML: Bone marrow lesion. †Cartilage defect: full-thickness cartilage defect denuded to subchondral bone. ‡SD: Standard deviation. §95%CI: 95% Confidence Interval.

**Supplementary Table 8.** Individual participant brace wear time (8-week average), KOOS pain change, and responder status (n=21), ordered by wear time.

| **Participant** | **Mean weekly wear**  **time (hours)** | **Change in**  **KOOS pain** | **Responder**  **(≥MCID)** |
| --- | --- | --- | --- |
| P17 | 76.3 | 5.6 | No |
| P18 | 75.3 | 0.0 | No |
| P10 | 73.3 | 22.2 | Yes |
| P21 | 71.4 | 2.8 | No |
| P12 | 68.9 | 16.7 | Yes |
| P09 | 57.1 | 27.8 | Yes |
| P13 | 55.3 | 30.6 | Yes |
| P19 | 51.8 | 0.0 | No |
| P07 | 51.5 | 13.9 | Yes |
| P06 | 50.0 | 5.6 | No |
| P02 | 49.3 | 11.1 | Yes |
| P03 | 48.3 | 19.4 | Yes |
| P20 | 46.4 | 16.7 | Yes |
| P16 | 46.0 | 27.8 | Yes |
| P01 | 41.1 | 11.1 | Yes |
| P15 | 26.8 | 19.4 | Yes |
| P08 | 21.4 | 41.7 | Yes |
| P05 | 13.4 | 25.0 | Yes |
| P04 | 11.5 | 50.0 | Yes |
| P11 | 0.0 | 22.2 | Yes |
| P14 | *Not recorded* | 38.9 | Yes |

*Wear time was significantly negatively correlated with change in KOOS pain (r = −0.59, p = 0.006, n = 20), robust to exclusion of the participant with zero recorded wear time, P11 (r = −0.64, p = 0.003, n = 19). Every non-responder (P17, P18, P19, P21, P06) had moderate-to-high wear time (50–76 hours/week); none had low wear time.
